# Influence of weight and type of planting material on fruit quality and its heterogeneity in pineapple [*Ananas comosu*s (L.) Merrill]

**DOI:** 10.3389/fpls.2014.00798

**Published:** 2015-01-21

**Authors:** V. Nicodème Fassinou Hotegni, Willemien J. M. Lommen, Euloge K. Agbossou, Paul C. Struik

**Affiliations:** ^1^Crop Physiology, Centre for Crop Systems Analysis, Wageningen UniversityWageningen, Netherlands; ^2^Faculté des Sciences Agronomiques, Université d'Abomey CalaviCotonou, Benin

**Keywords:** *Ananas comosus*, cultural practices, hapas, suckers, slips, heterogeneity, uniformity, variation

## Abstract

Cultural practices can affect the quality of pineapple fruits and its variation. The objectives of this study were to investigate (a) effects of weight class and type of planting material on fruit quality, heterogeneity in quality and proportion and yield of fruits meeting European export standards, and (b) the improvement in quality, proportion and yield of fruits meeting export standards when flowering was induced at optimum time. Experiments were conducted in Benin with cvs Sugarloaf (a Perola type) and Smooth Cayenne. In cv. Sugarloaf, experimental factors were weight class of planting material (light, mixed, heavy) and time of flowering induction (farmers', optimum) (Experiment 1). In cv. Smooth Cayenne an additional experimental factor was the type of planting material (hapas, ground suckers, a mixture of the two) (Experiment 2). Fruits from heavy planting material had higher infructescence and fruit weights, longer infructescences, shorter crowns, and smaller crown: infructescence length than fruits from light planting material. The type of planting material in Experiment 2 did not significantly affect fruit quality except crown length: fruits from hapas had shorter crowns than those from ground suckers. Crops from heavy planting material had a higher proportion and yield of fruits meeting export standards than those from other weight classes in Experiment 1 only; also the type of planting material in Experiment 2 did not affect these variates. Heterogeneity in fruit quality was usually not reduced by selecting only light or heavy planting material instead of mixing weights; incidentally the coefficient of variation was significantly reduced in fruits from heavy slips only. Heterogeneity was also not reduced by not mixing hapas and ground suckers. Flowering induction at optimum time increased the proportion and yield of fruits meeting export standards in fruits from light and mixed slip weights and in those from the mixture of heavy hapas plus ground suckers.

## Introduction

Several recent reports stress the low export volume of fruits from developing countries to international markets (Subramanian and Matthijs, 2007; Van Melle and Buschmann, 2013). This low export volume is due to the poor average quality of the fruits as well as the low uniformity in fruit quality (Joosten, 2007; Temu and Marwa, 2007; Van Melle et al., 2013). This is also the case for pineapple [*Ananas comosus* (L.) Merrill] from Benin (Fassinou Hotegni et al., 2014a), where pineapple yield is high but the quality is poor and heterogeneous. Improvement of both average and uniformity in quality is crucial to improve the marketability of the produce. Since pineapple quality can hardly be improved after harvesting fruits, this study concentrates on improving cultural practices at early and later crop stages.

In pineapple cultivation, the type and weight of planting material may affect average fruit quality as well as the uniformity in fruit quality attributes. The planting material consists of different types of side shoots sourced from plants kept in the field after fruit harvest: *slips* (side shoots produced on the peduncle at the base of the fruit), *hapas* (side shoots produced above ground on the stem at the junction of the stem and the peduncle), and *suckers* (side shoots originating on the stem; *ground suckers* originate below ground on the stem) (Hepton, 2003). Their appearance and number depend on the pineapple cultivar (Norman, 1976). At planting, pineapple producers in Benin often mix different types and weights of planting material, depending on their availability. It is well-known that larger or heavier planting material shows more vigorous growth than smaller or lighter planting material (e.g., Norman, 1976; Reinhardt et al., 2003) and would produce more vigorous plants at flowering induction time than smaller or lighter planting material. Higher plant vigor at flowering induction is associated with higher fruit (defined as infructescence + crown) and infructescence weights, a lower crown weight and crown length and consequently a lower ratio crown: infructescence length (Fassinou Hotegni et al., 2014b). In this paper, we hypothesize that heavy planting material will produce more vigorous plants at flowering induction and consequently will yield higher average fruit quality than light planting material.

Mixing different weights within the same type of planting material may therefore increase the heterogeneity in plant vigor and may give more variable fruit quality than would be the case in crops originating from a narrow range of planting material weights. Mixing different types of planting material may also lead to a higher heterogeneity in plant vigor than in crops originating from the same type of planting material and consequently may give more variable fruits. Many authors claimed the need to have uniform planting material at planting time (Reinhardt et al., 2000; Hepton, 2003) but information on the effect of uniformity of planting material on average fruit quality and its heterogeneity is lacking. In this paper, we hypothesize that using (1) a narrow weight range within the same type of planting material at planting time and (2) only one type of planting material leads to more uniform fruit quality at harvest compared to mixing different weights and types of planting material.

In pineapple cultivation in Benin, artificial flowering induction of pineapple crops takes place 9–13 months after planting. Plants are therefore induced to flower regardless of whether they originate from mixtures of different weights and types of planting material or not. In this paper we hypothesize that flowering induction at optimum induction time, i.e., the moment when most plants within each planting material type/weight interval are well-developed and capable to yield marketable fruits, would improve average fruit quality and increase the proportion and yield of fruits exportable to international markets compared to farmers' flowering induction time.

The objectives of this research were to evaluate the effects of weight, type, and mixtures of different weights and types of planting material on the average fruit quality, heterogeneity in fruit quality, and the proportion and yield of fruits meeting the criteria for European standard. We also aimed at studying if flowering induction at the optimum time increases the average fruit quality and proportion and yield of fruits meeting the export criteria when compared to flowering induction at farmers' time.

## Materials and methods

### Experimental sites and cultural practices

Two experiments were carried out in the Atlantic department in the south of Benin between November 9, 2011 and September 20, 2013 with cvs Sugarloaf (Experiment 1) and Smooth Cayenne (Experiment 2). Cv. Sugarloaf is a Perola pineapple type grown by 97% of the pineapple producers in the department and is known to produce numerous slips; hence slips are the common planting material used for its propagation. In cv. Smooth Cayenne, mixtures of hapas and ground suckers are commonly used for planting; the fruits of cv. Smooth Cayenne are exported to European markets (Fassinou Hotegni et al., 2012). The mean monthly temperatures varied between 24.9 and 29.3°C during the experiments with the lowest mean temperature recorded in August 2012 and the highest mean temperature in March 2012 and 2013. The total rainfall was 2346 mm during the experiment with cv. Sugarloaf and 2142 mm during the experiment with cv. Smooth Cayenne. Information on the field locations and cultural practices (all practices except flowering induction and harvesting times) is presented in Table 1.

**Table 1:** **Field information and cultural practices in the two experiments with cvs Sugarloaf or Smooth Cayenne**.

	**Experiment 1, Cv. Sugarloaf**	**Experiment 2, Cv. Smooth Cayenne**
Location	06°36′10.8″N and 02°16′58.1″E	06°33′21.2″N and 02°14′47.8″E
Municipality (district)	Zè (Tangbo Djevie)	Tori Bossito (Lankoutan)
Soil type	Ferralitic soil	Idem
Climate	Subequatorial	Idem
Planting time	24 February 2012	9 November 2011
Types of planting material used	Slips	Hapas and ground suckers
Planting material treatment before planting	Sorting in different weight classes	Idem
Planting arrangement	Flat beds of two alternating rows	Idem
Plant spacing: BP^a^ × BR^b^/BDR^c^ (cm)	35 × 40/70	40 × 45/80
Plant density (plants/m^2^)	5.19	4.00
First Urea (46N) + NPK (10-20-20) application	2 MAP^d^ (30 April 2012)	5–6 MAP (23 April 2012)
*Application form*	Solid at the base of the plants	Idem
*Dose per plant (g Urea + g NPK)*	6 + 3	Idem
Second Urea (46N) + NPK (10–20–20) application	5 MAP (20 July 2012)	8 MAP (14 July 2012)
*Application form*	Solid at the base of the plants	Idem
*Dose per plant (g Urea + g NPK)*	6 + 3	Idem
Third Urea (46N) + NPK (10-20-20) application	Not applied	10 MAP (06 September 2012)
*Application form*		Solid at the base of the plants
*Dose per plant (g Urea + g NKP)*		3 + 6
Weed control	Hand weeding	Idem

a *BP, spacing between plants within a row*.

b *BR, spacing between rows*.

c *BDR, spacing between double rows*.

d *MAP, months after planting*.

### Experimental design and treatments

In the experiment with cv. Sugarloaf, a split-plot design was used with four replications: *flowering induction time* was the main factor and had two levels: flowering induction following farmers' practice and flowering induction at the optimum time (See Section Flowering Induction Practice); *weight class of the planting material* (slips were the only planting material used) was the split factor and had three levels: light planting material with a narrow interval [100–325] g; heavy planting material with a narrow interval [325–550] g and a mixture of planting material from the two previous intervals in the proportion half [100–325] g and half [325–550] g. In the experiment with cv. Smooth Cayenne a split-split-plot design was used with four replications: *flowering induction time* was the main factor and had two levels: flowering induction following farmers' practice and flowering induction at the optimum time (See Section Flowering Induction Practice); *type of planting material* was the sub factor and had three levels: hapas, ground suckers, and a mixture of hapas and ground suckers; *weight class of the planting material* was the sub-sub factor and had three levels: light planting material with a narrow interval [125–400] g; heavy planting material with a narrow interval [400–675] g and a mixture of planting material from the two previous intervals in the proportion half of each for the single planting material types. For the mixture of planting material types, i.e., hapas and ground suckers, proportions used were 75% hapas and 25% ground suckers (reflecting the farmers' practice in the mixture of the different types of cv. Smooth Cayenne planting material) except for the mixture of both the weights and types planting material where the ratio 67% hapas and 33% ground suckers was used.

**Figure 1:**
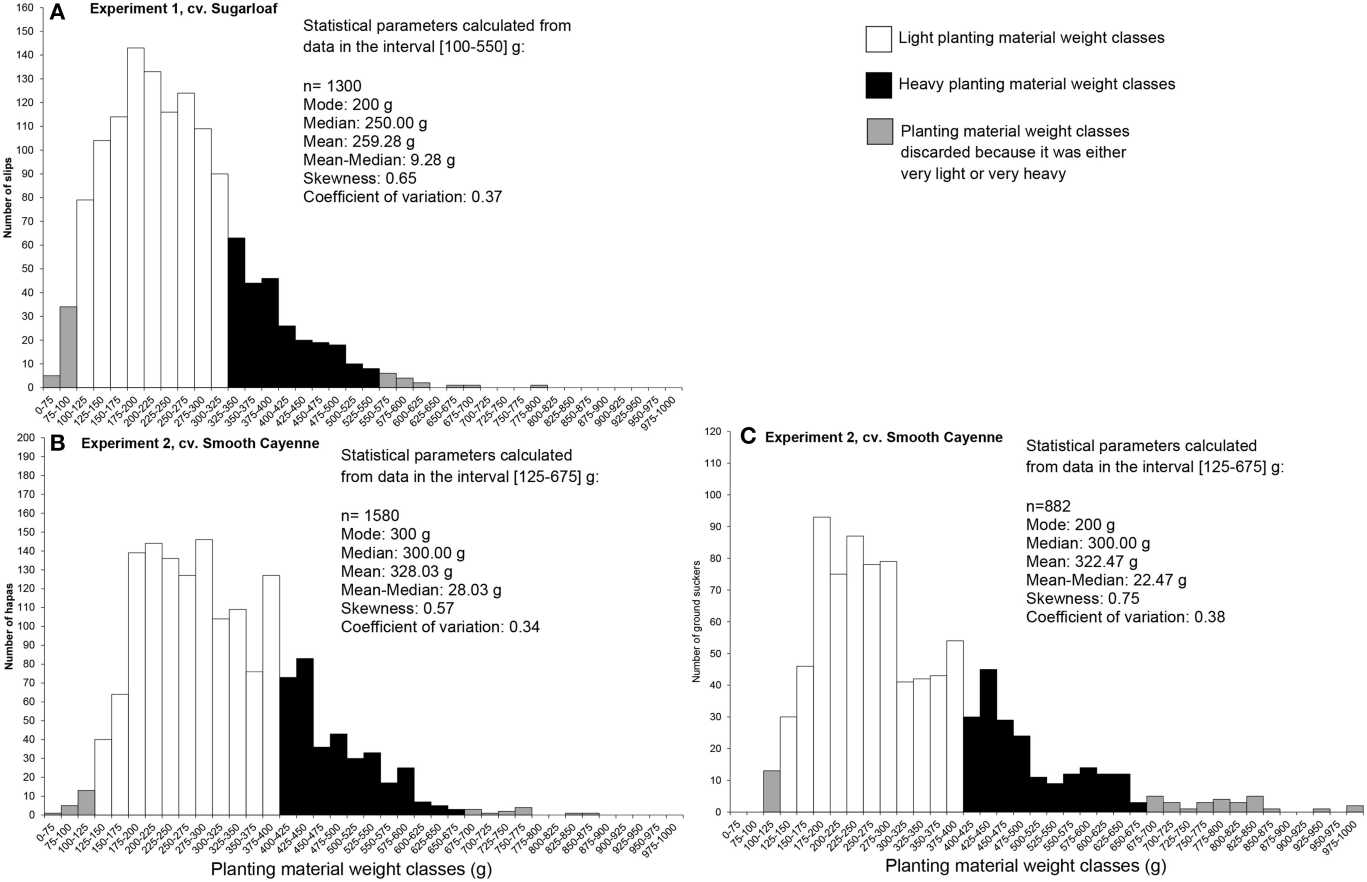
**Frequency distribution of the planting material weights in the lots from which the classes used in Experiments 1 and 2 were derived. (A)** Slips (Experiment 1); **(B)** Hapas (Experiment 2); and **(C)** Ground suckers (Experiment 2).

The planting material was collected in farmers' fields from harvested plants. The lower and upper limit of the light and heavy planting material intervals in the experiments were derived from weighing 1320 slips in cv. Sugarloaf and 1598 hapas and 910 ground suckers in cv. Smooth Cayenne. The very light and very heavy planting material were discarded. Within each planting material lot, the light planting material was most abundant as shown by a positive skewness for all three types of planting material (Figure 1). All planting material lots were variable with a coefficient of variation (CV) between 0.34 and 0.38 across the classes used in the experiments.

In both experiments, each net plot consisted of 60 plants arranged in 6 lines of 10 plants each. The net plots were surrounded by at least two guard rows and two guard plants in a row.

### Flowering induction practice

Flowering induction was carried out by means of carbide of calcium (CaC_2_), a compound producing acetylene when it reacts with water. Using farmers' practices, 50 ml of a solution containing 10 g/l and 15 g/l of CaC_2_ for Sugarloaf and Smooth Cayenne, respectively, was applied into the center of the leaf rosette in each plant. This application was carried out once in cv. Sugarloaf and three times, with intervals of 3 days, in cv. Smooth Cayenne. Farmers induce flowering between 9 and 13 months after planting (Fassinou Hotegni et al., 2012). In the present experiments, flowering induction time according to farmers' practice was 12 months after planting. The optimum time for flowering induction was defined as the moment when 75% of the plants of a specific treatment showed a plant vigor expressed as the product of the number of functional leaves × the D-leaf length (the longest leaf on the pineapple plant) that was higher or equal to 1235 leaf.cm for cv. Sugarloaf and 2300 leaf.cm for cv. Smooth Cayenne. These values of the product in the two pineapple cultivars were based on recent experiments by Fassinou Hotegni et al. (2014b) that indicated that fruit weight for export of pineapple to European markets were met for plants within a crop when the product of the number of functional leaves × the D-leaf length reached at least 1235 leaf.cm in cv. Sugarloaf and 2300 leaf.cm in cv. Smooth Cayenne.

Following farmers' practices (Fassinou Hotegni et al., 2012), maturity was only induced artificially in cv. Smooth Cayenne by spraying 3.5 ml of a solution of 14 ml/l Ethephon (2-chloroethylphosphonic acid), a compound producing ethylene, on the skin of each fruit. The application was carried out at 143 days after flowering induction and repeated 4 days later. The fruits were harvested following farmers' practice which was 7 days after the last application of Ethephon in cv. Smooth Cayenne. In cv. Sugarloaf, the harvesting time was when the skin color had started to change from green to yellow in at least 25% of the plants in a net plot. All fruits in that plot were harvested on that day and were individually processed.

Information on the flowering induction and harvesting times of the different treatments is summarized in Table S1 in the Supplementary Material (Data sheet 1).

### Data collected

Data on the plant development at flowering induction included the number of functional leaves and the D-leaf length collected per plant 1 week before flowering induction in the plots induced at the farmers' flowering induction time. The product of both was computed. In the plots to be induced at the optimum flowering induction time, the number of functional leaves and the D-leaf length were collected from 10 months after planting until they were induced. The product of both was computed to determine the optimum flowering induction time following the criteria set for the optimum flowering induction time for each pineapple cultivar.

Data on the fruit quality included: weights and lengths of the fruit (infructescence + crown) and the infructescence and crown separately, the ratio of crown: infructescence length, percentage of flesh translucency, internal browning, and total soluble solids concentration (TSS) in the fruit juice. The weight and length attributes, and the TSS were determined following the procedures described by Fassinou Hotegni et al. (2014b). The percentage of fruit with translucent flesh and internal browning was determined following the methods of Paull and Reyes (1996). Minimum quality criteria for fruits to be European export standards include: the fruit weight should be between 0.70 and 2.75 kg, the ratio crown: infructescence length should be between 0.5 and 1.5 and TSS should be at least 12° Brix (Codex Alimentarius, 2005). These criteria were used to compute the percentage of exportable pineapple fruits per treatment.

### Data analysis

Data were analyzed using GenStat for Windows 16th Edition (VSN International, 2013). Effects of weight class and type of planting material were only analyzed for the treatments in which flowering was induced 12 months after planting, i.e., at farmers' flowering induction time. Therefore, a One-Way ANOVA was used in Experiment 1 for studying effects of weight class and a Two-Way ANOVA for split plot was used in Experiment 2 for studying effects of weight class and type. Before analysis, the data on the percentage translucent flesh were transformed using square root transformation (*x* + 0.5)^1/2^ (Bartlett, 1936; Gonzalez, 2009). The heterogeneity in fruit quality attributes was described using two variation parameters: the CV and the range 5–95%. Focus was on the agronomically relevant variation parameter, i.e., the CV, as used by Michaels et al. (1988) to establish variation in seed size and by Woodward (2007) to establish variation in kiwifruit quality. The range 5–95% is presented for detailed understanding. Data on the proportion of fruits meeting the minimum European market criteria for pineapple were transformed using arcsine transformation on the square root of the proportion before analysis (Fernandez, 1992). Proportions equal to 0 or 1 were replaced by (1/4n) and [1 – (1/4n)], respectively, where n is the total number of fruits per net plot (Fernandez, 1992). Data on the yield of fruits meeting the criteria for European standards were transformed using natural-log-transformation before analysis (Field, 2009). Means were separated using the LSD test, with different LSD values being necessary for comparisons between means within and across different types of planting material in Experiment 2 due to its split-plot design.

To compare the average fruit quality and proportion and yield of exportable fruits at farmers' induction time with those at optimum flowering induction time a *t*-test was carried out for the individual planting material treatments. Differences between harvest times are reported as well as their significance.

## Results

### Effects of weight and type of planting material on average and variation in plant vigor at farmers' flowering induction time

**Figure 2:**
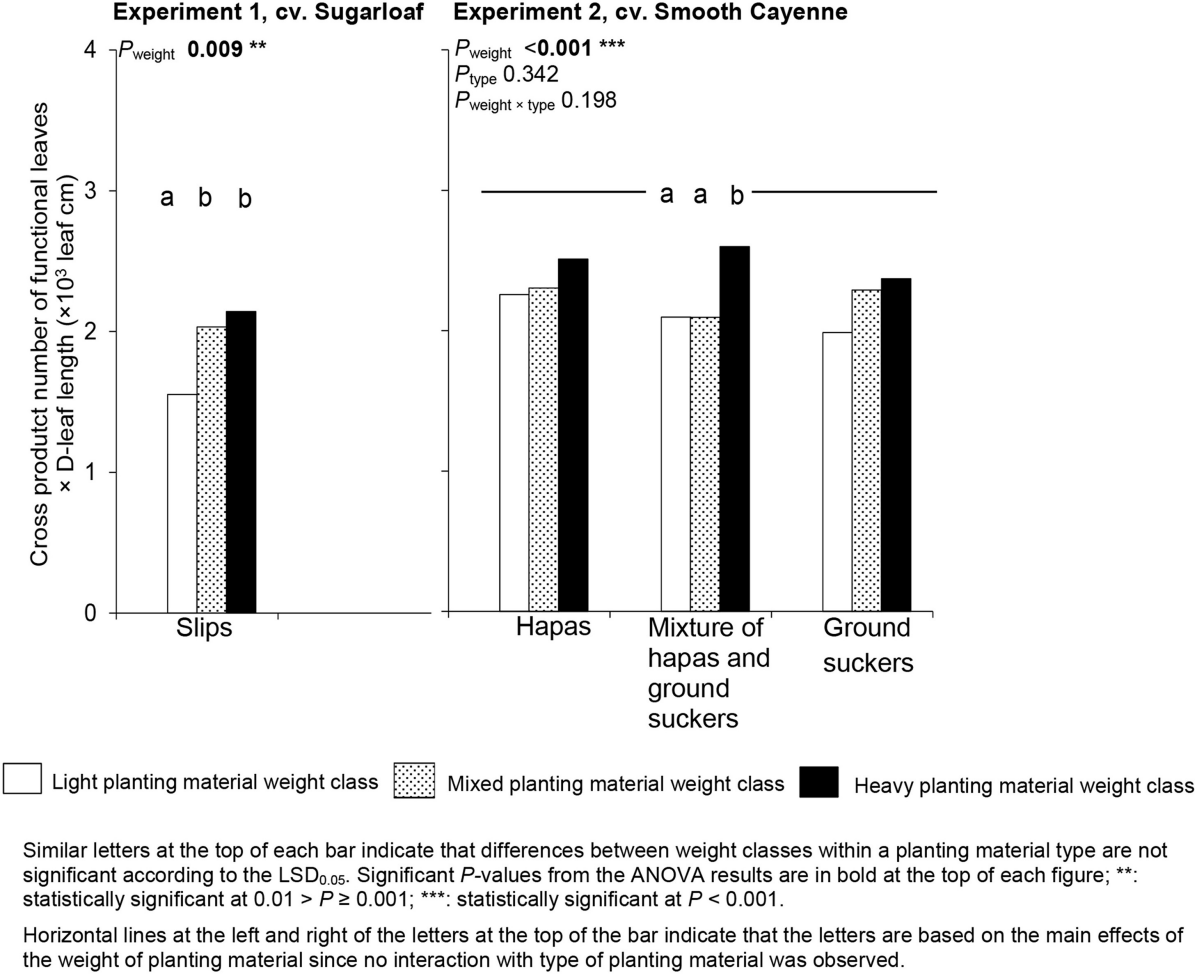
**Average plant vigor (the product number of functional leaves × D-leaf length) at farmers' flowering induction time as affected by weight and type of planting material in Experiments 1 and 2**.

In both experiments, heavy planting material resulted in more vigorous plants than light planting material at the farmers' flowering induction time (Figure 2). In Experiment 1, the mixture of planting material weights gave more vigorous plants than light planting material, but did not differ in vigor from plants from heavy planting material (Figure 2). In Experiment 2, plants from the mixture of planting material weights did not differ significantly in vigor from plants from light planting material, but had a lower vigor than plants from heavy planting material (Figure 2).

**Figure 3:**
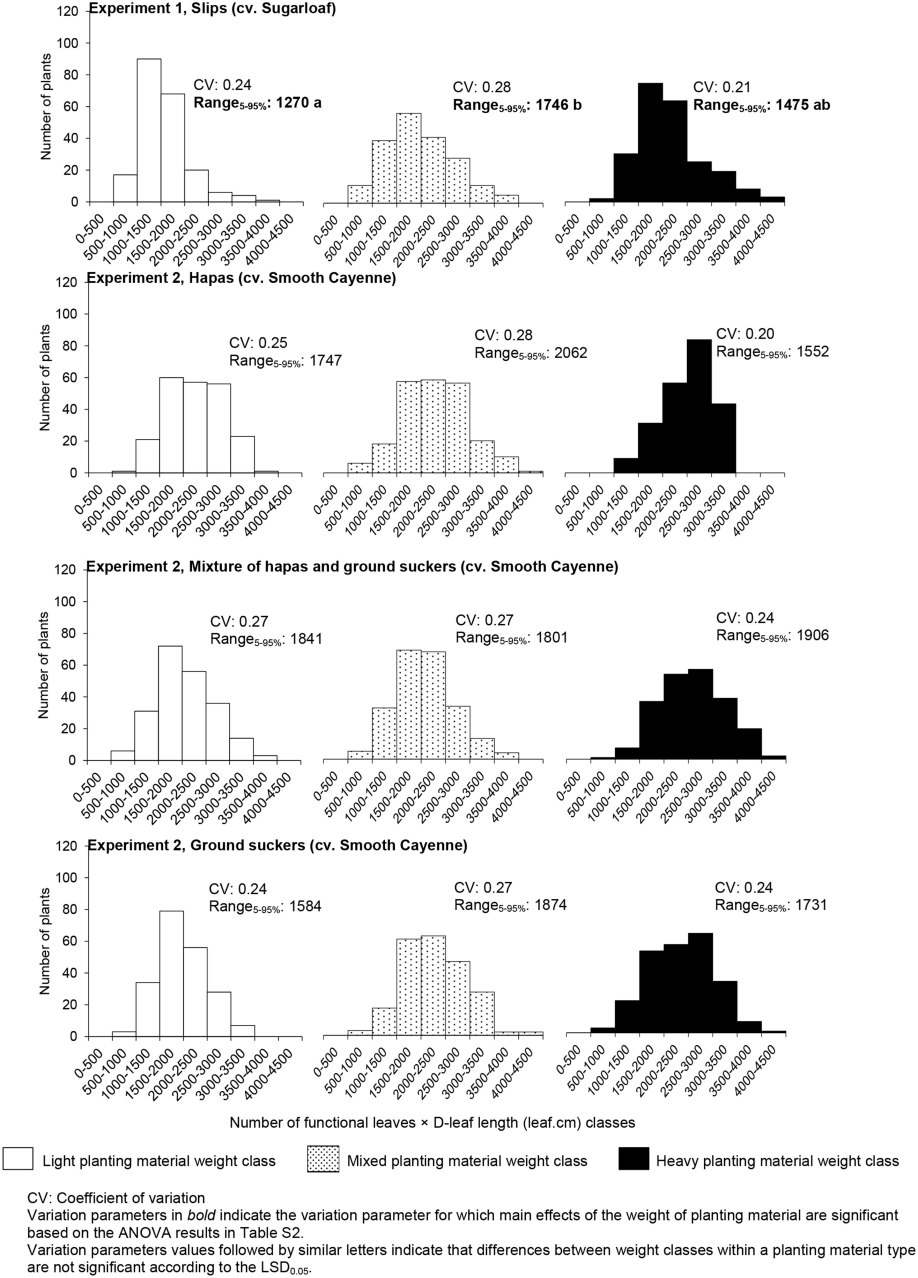
**Frequency distribution of plant vigor (the product number of functional leaves × D-leaf length) in plants induced at farmers' flowering induction time and its variation (expressed in different variation parameters) as affected by the planting material weight and type**.

The weight class of planting material had no significant effect on the CV in vigor of the individual plants at the induction time (Table 2), but plants from the mixed weight class had a higher range 5–95% in vigor than plants from light planting material in Experiment 1, whereas plants from the heavy planting material class did not differ significantly from any of these classes in this variate (Figure 3). In Experiment 2, the weight class had no effect on CV and range 5–95% in the vigor of plants at induction time (Table 2).

**Table 2:** ***P*-values for the effects of weight and type of planting material and their interaction on variation (expressed in different variation parameters) in vigor of individual plants at farmers' flowering induction time, in Experiments 1 and 2**.

**Variation parameter and factor**	**Experiment 1, cv. Sugarloaf (Slips)**	**Experiment 2, cv. Smooth Cayenne (Hapas, ground suckers, and mixture of both)**
**COEFFICIENT OF VARIATION IN VIGOR OF INDIVIDUAL PLANTS**		
Weight of planting material (weight)	0.065	0.183
Type of planting material (type)	–^a^	0.599
Weight × type	–	0.875
**RANGE 5–95% IN VIGOR OF INDIVIDUAL PLANTS**		
Weight of planting material (weight)	0.035^*^	0.433
Type of planting material (type)	–	0.283
Weight × type	–	0.597

*Vigor was assessed as the product of the number of functional leaves × the D-leaf length*.

* Significant at the 0.05 probability level;

a *not applicable because type of planting material was not a factor in this experiment. Values in bold indicate the P-value of the effect (main or interaction) considered to draw conclusions in the text*.

In Experiment 2 where the differences between ground suckers, hapas, and their mixture were studied, the type of planting material had no significant effect on average plant vigor at farmers' flowering induction time (Figure 2) nor on the variation in plant vigor for both variation parameters (Table 2).

### Effects of weight of planting material on average fruit quality attributes

**Figure 4:**
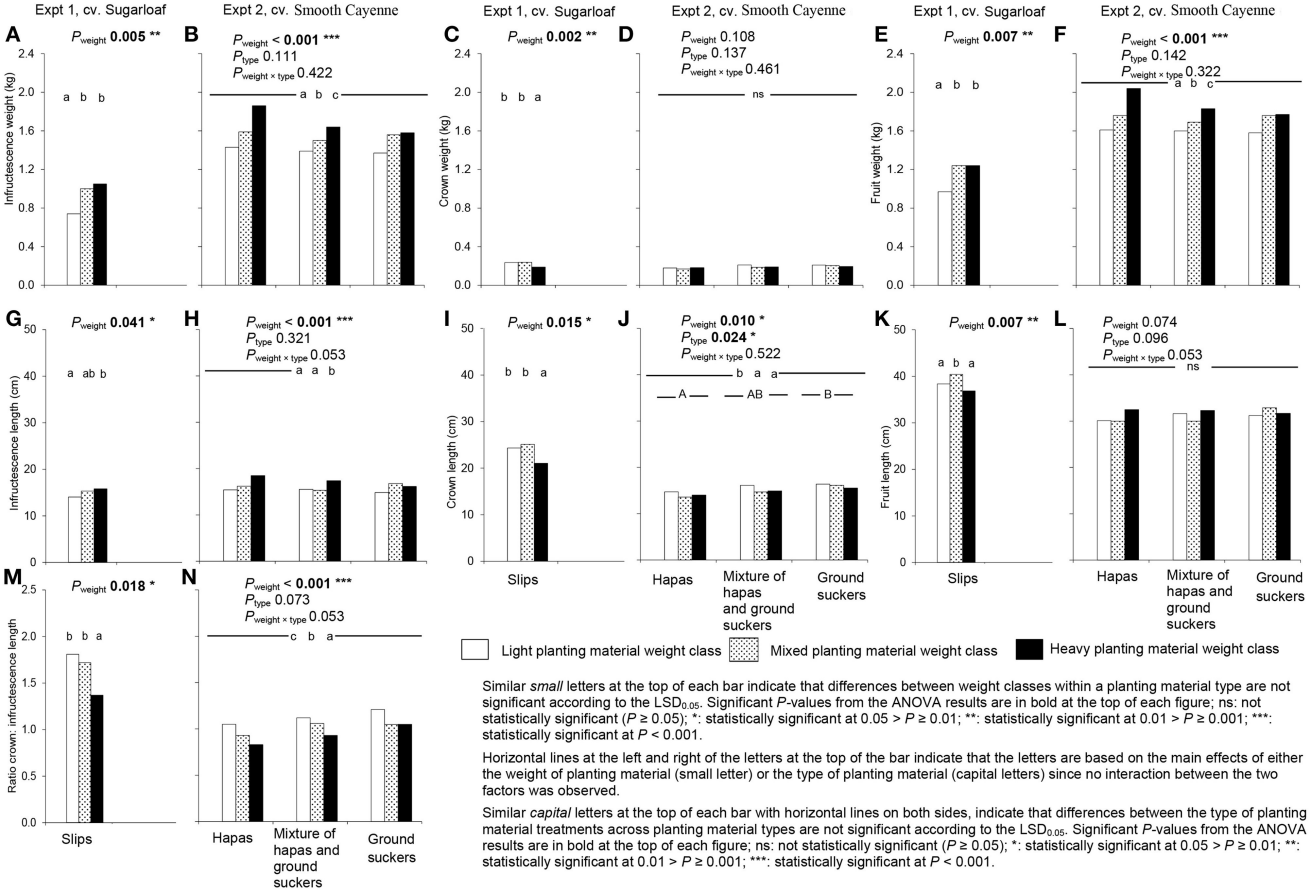
**Effect of weight and type of planting material on average fruit weights attributes (A–F) and average fruit length attributes (G–N) in plants induced at farmers' flowering induction time in Experiments 1 (A,C,E,G,I,K,M) and 2 (B,D,F,H,J,L,N)**.

Fruits from heavy planting material had higher infructescence and fruit weights than fruits from light planting material in both experiments and all types of planting material (Figures 4A,B,E,F). In Experiment 1, fruits from mixed slip weights had higher infructescence and fruit weights than fruits from light planting material, but did not differ significantly from those from heavy planting material (Figures 4A,E). In Experiment 2, the infructescence and fruit weights of plants from the mixtures of planting material weights were intermediate between those from the light and heavy planting material (Figures 4B,F). An effect of planting material weight on the crown weight was only observed in Experiment 1 where fruits from light slips and mixed slip weights did not differ in crown weight, but had heavier crowns than fruits from heavy slips (Figure 4C).

Fruits from heavy planting material had a taller infructescence, a shorter crown and smaller crown: infructescence length than those from light planting material in both experiments and all types of planting material (Figures 4G–J,M,N); however, there were no significant differences in total fruit length between light and heavy planting material. Fruits from mixed and light planting material did not differ in infructescence length in both experiments (Figures 4G,H) and in crown length in Experiment 1 (Figure 4I); in Experiment 2, crown length of fruits from mixed planting material did not differ from that from heavy planting material (Figure 4J). The crown: infructescence length in fruits from mixed slip weights did not differ significantly from light slips, but was higher than in fruits from heavy slips in Experiment 1 (Figure 4M); in Experiment 2, the ratio crown: infructescence length of fruits from mixed planting material was intermediate between the ratio's in fruits from light and heavy planting material (Figure 4N). An effect on fruit length was found in Experiment 1 only; fruits from mixed slip weights had a higher fruit length than fruits from heavy and light slips (Figure 4K).

**Figure 5:**
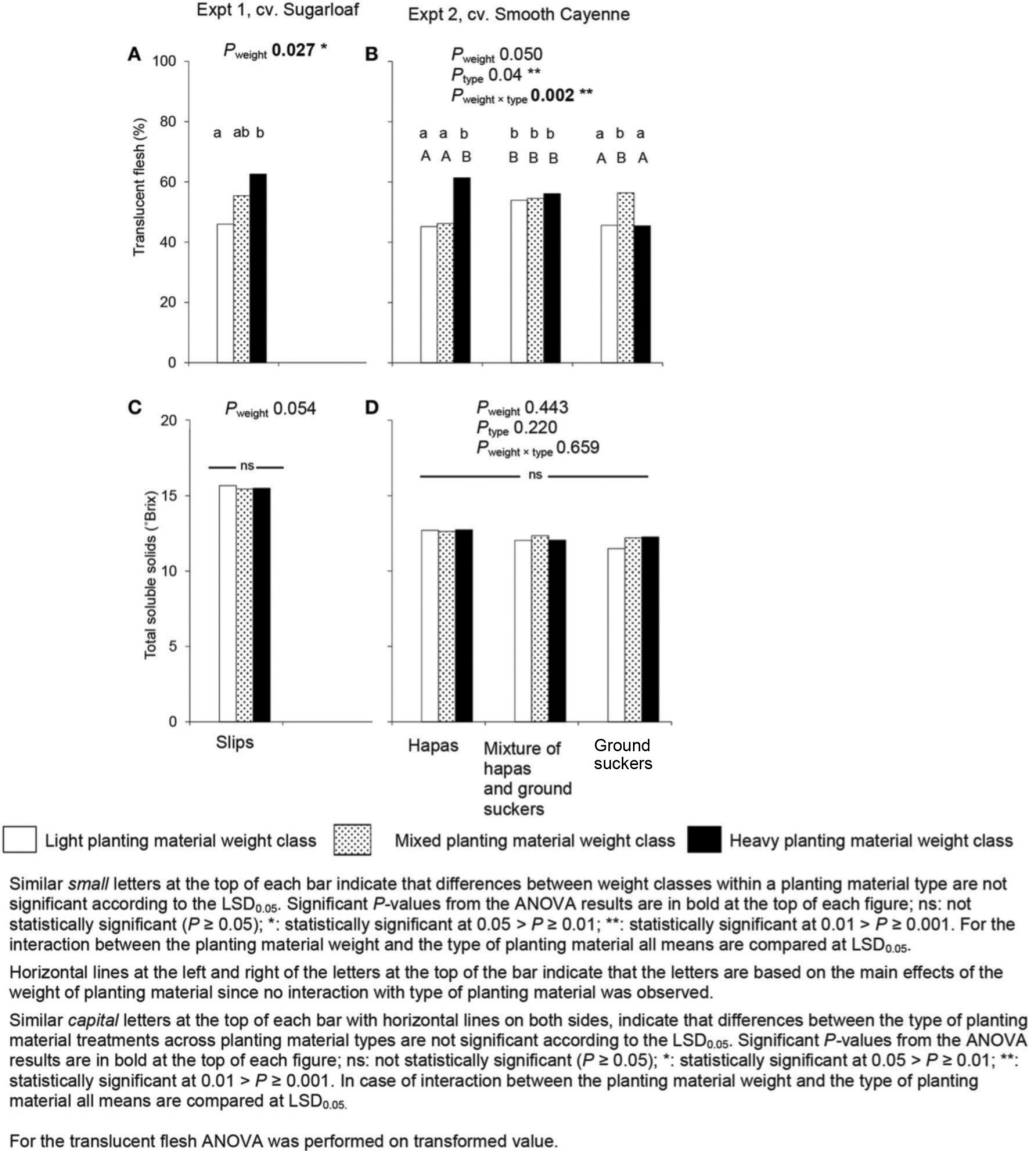
**Average translucent flesh (A,B) and total soluble solids (C,D) in fruits from plants induced at farmers' flowering induction time as affected by weight and type of planting material in Experiments 1 (A,C) and 2 (B,D)**.

The effect of planting material weight on the percentage translucent flesh was only clear in Experiment 1: fruits from heavy slips had a higher percentage translucent flesh than those from light slips (Figure 5A); fruits from mixed slip weights did not differ from those from light or heavy slips (Figure 5A).

The weight of the planting material had no effect on the TSS in any of the experiments (Figures 5C,D).

### Effects of type of planting material on average pineapple fruit quality attributes

The type of planting material as investigated in Experiment 2 had no significant effect on fruit weight attributes (Figures 4B,D,F), and among fruit length attributes only on crown length: fruits originating from hapas had shorter crowns than those originating from ground suckers (Figure 4J).

An effect of the type of planting material was observed on the percentage translucent flesh in Experiment 2 (Figure 5B), but the effect was not clear enough to draw an unambiguous conclusion. There was no effect of the type of planting material on TSS (Figure 5D).

### Effects of weight and type of planting material on variation in fruit quality attributes

The weight class of the planting material had significant effects on the CV in crown weight and infructescence length in Experiment 1 and fruit length in Experiment 2 (Table 3), but not on the CV of the other quality attributes.

**Table 3:** ***P*-values for the effects of weight and type of planting material and their interaction on fruit-to-fruit variation (expressed as CV and range 5–95%) in different fruit quality attributes of individual fruits in Experiments 1 and 2**.

**Variation parameter and factor**	**Experiment 1, cv. Sugarloaf (Slips)**		**Experiment 2, cv. Smooth Cayenne (Hapas, ground suckers, and mixture of hapas and ground suckers)**	
	**CV^a^**	**5–95%**	**CV**	**5–95%**
**INFRUCTESCENCE WEIGHT (KG)**				
Weight of planting material (Weight)	0.053	0.106	0.107	0.170
Type of planting material (Type)	–^b^	–	0.412	0.382
Type × Weight	–	–	0.382	0.573
**CROWN WEIGHT (KG)**				
Weight of planting material (Weight)	0.012^*^	0.189	0.487	0.233
Type of planting material (Type)	–	–	0.675	0.011^*^
Type × Weight	–	–	0.137	0.490
**FRUIT WEIGHT (KG)**				
Weight of planting material (Weight)	0.128	0.106	0.130	0.266
Type of planting material (Type)	–	–	0.701	0.182
Type × Weight	–	–	0.374	0.696
**INFRUCTESCENCE LENGTH (CM)**				
Weight of planting material (Weight)	0.021^*^	0.087	0.164	0.482
Type of planting material (Type)	–	–	0.606	0.497
Type × Weight	–	–	0.941	0.956
**CROWN LENGTH (CM)**				
Weight of planting material (Weight)	0.299	0.769	0.635	0.307
Type of planting material (Type)	–	–	0.708	0.030^*^
Type × Weight	–	–	0.145	0.179
**FRUIT LENGTH (CM)**				
Weight of planting material (Weight)	0.705	0.882	0.032^*^	0.340
Type of planting material (Type)	–	–	0.461	0.268
Type × Weight	–	–	0.863	0.907
**RATIO CROWN: INFRUCTESCENCE LENGTH**				
Weight of planting material (Weight)	0.304	0.005^**^	0.655	0.034^*^
Type of planting material (Type)	**–**	**–**	0.462	0.415
Type × Weight	–	–	0.439	0.782
**TRANSLUCENT FLESH (%)**				
Weight of planting material (Weight)	0.626	0.528	0.565	0.603
Type of planting material (Type)	–	**–**	0.181	0.379
Type × Weight	–	–	0.263	0.509
**TOTAL SOLUBLE SOLIDS (° BRIX)**				
Weight of planting material (Weight)	0.751	0.929	0.792	0.590
Type of planting material (Type)	–	**–**	0.020^*^	0.139
Type × Weight	–	–	0.539	0.551

* *Significant at the 0.05 probability level*.

** *Significant at the 0.01 probability level*.

a *Coefficient of variation*.

b *Not applicable because type of planting material was not a factor in this experiment. Values in bold indicate the P-value of the effect (main or interaction) considered to draw conclusions in the text and **Table 4***.

In Experiment 1, fruits from heavy slips had a higher CV in crown weight (Table 4) and a lower CV in infructescence length (Table 4) than fruits from mixed and light slips. Fruits from mixed and light slips did not differ in CV in crown weight and infructescence length (Table 4). In Experiment 2, fruits from heavy planting material had a lower CV in fruit length than fruits from mixed and light planting material (Table 4). Plants from mixed and light slips did not differ in the CV in fruit length (Table 4).

**Table 4:** **Fruit-to-fruit variation (expressed as CV and range 5–95%) in different quality attributes in plants induced at farmers' flowering induction time in Experiments 1 and 2**.

**Fruit quality attribute, experiment and treatment**	**Variation parameters**	
	**CV^a^**	**5–95%**
**INFRUCTESCENCE WEIGHT (KG)**		
Expt 1, cv. Sugarloaf	0.33	0.95
Expt 2, cv. Smooth Cayenne	0.39	1.88
**CROWN WEIGHT (KG)**		
Expt 1, cv. Sugarloaf	0.25	0.16
Light slips	**0.23 a**^b^	
Mixture of weights	**0.20 a**	
Heavy slips	**0.30 b**	
Expt 2, cv. Smooth Cayenne	0.39	0.21
Hapas		**0.20 a**
Mixture of types		**0.22 b**
Ground suckers		**0.22 b**
**FRUIT WEIGHT (KG)**		
Expt 1, cv. Sugarloaf	0.28	1.01
Expt 2, cv. Smooth Cayenne	0.35	1.85
**INFRUCTESCENCE LENGTH (CM)**		
Expt 1, cv. Sugarloaf	0.17	8.17
Light slips	**0.19 b**	
Mixture of weights	**0.18 b**	
Heavy slips	**0.14 a**	
Expt 2, cv. Smooth Cayenne	0.22	11.28
**CROWN LENGTH (CM)**		
Expt 1, cv. Sugarloaf	0.15	11.52
Expt 2, cv. Smooth Cayenne	0.37	15.24
Hapas		**14.56 a**
Mixture of types		**15.44 b**
Ground suckers		**15.74 b**
**FRUIT LENGTH (CM)**		
Expt 1, cv. Sugarloaf	0.11	13.11
Expt 2, cv. Smooth Cayenne	0.17	15.97
Light planting material	**0.18 b**	
Mixture of weights	**0.17 b**	
Heavy planting material	**0.15 a**	
**RATIO CROWN: INFRUCTESCENCE LENGTH**		
Expt 1, cv. Sugarloaf	0.26	1.36
Light slips		**1.63 b**
Mixture of weights		**1.44 b**
Heavy slips		**1.01 a**
Expt 2, cv. Smooth Cayenne	0.51	1.55
Light planting material		**1.66 b**
Mixture of weights		**1.60 ab**
Heavy planting material		**1.40 a**
**TRANSLUCENT FLESH (%)**		
Expt 1, cv. Sugarloaf	0.55	81.50
Expt 2, cv. Smooth Cayenne	0.39	57.52
**TOTAL SOLUBLE SOLIDS (° BRIX)**		
Expt 1, cv. Sugarloaf	0.09	4.41
Expt 2, cv. Smooth Cayenne	0.12	4.09
Hapas	**0.09 a**	
Mixture of types	**0.12 b**	
Ground suckers	**0.11 b**	

*Effects of weight class or type of planting material are presented when significant (Table 3)*.

a *Coefficient of variation*.

b *Values in bold followed by the same letters within an attribute, are not significantly different according to the LSD-test (0.05)*.

The type of planting material had a significant effect on the CV in TSS in Experiment 2: fruits from hapas had a lower CV in TSS than fruits from ground suckers and mixed planting types, that did not differ from each other (Table 4).

Histograms picturing the variation in the quality attributes in the individual treatments are presented in Figures S1–S9 in the Supplementary Material (Data sheet 2).

### Effects of weight and type of planting material on percentage and yield of fruits meeting European export standards

**Figure 6:**
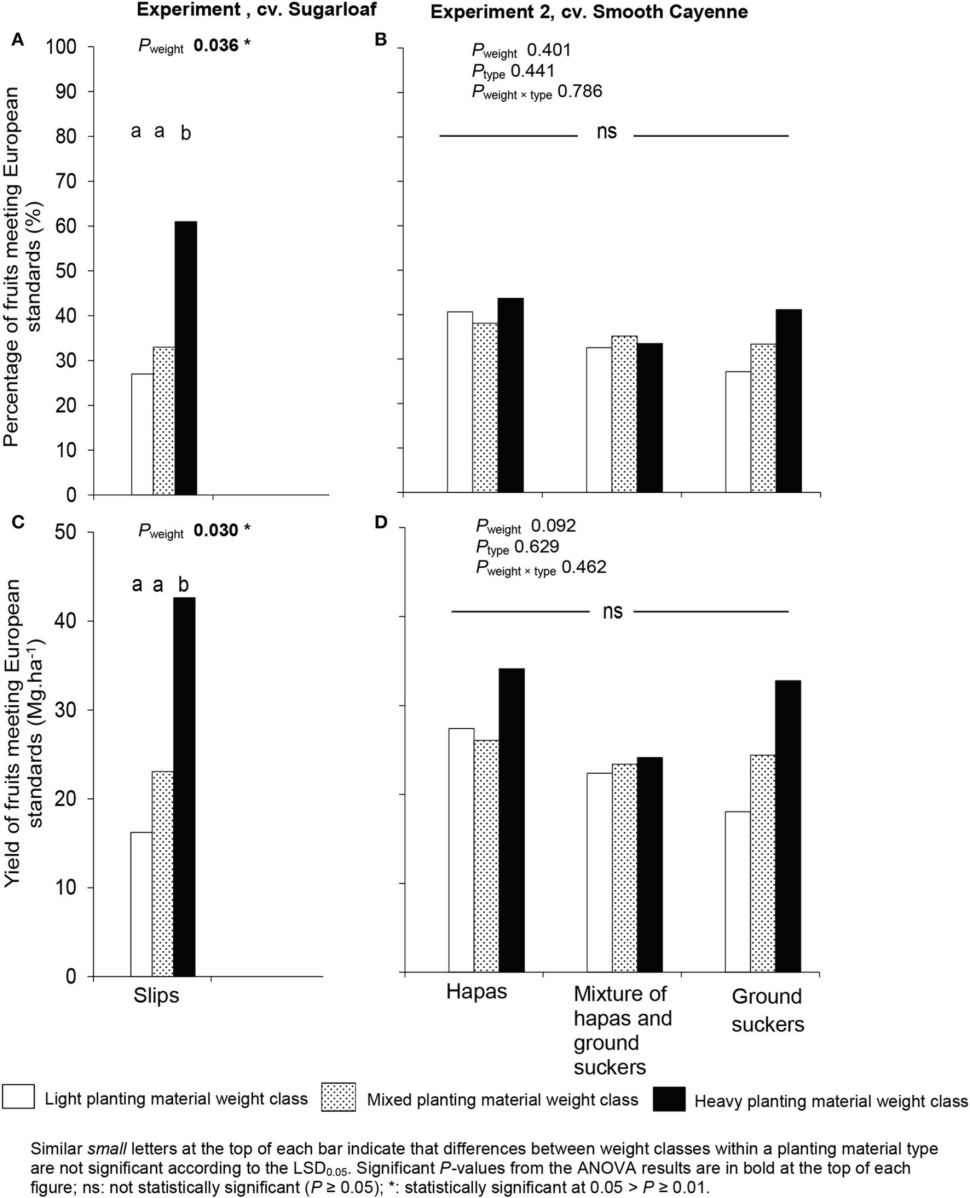
**Percentages of fruits meeting European export standards (A,B) and yield of fruits meeting European export standards (C,D) in the lot of fruits from plants induced at farmers' flowering induction time as affected by weight and type of planting material in Experiments 1 and 2**.

Effects of the planting material weight class on percentage and yield of fruits meeting export standards was found only in Experiment 1: plants from heavy slips gave a higher percentage of fruits meeting European export standards than plants from mixed and light slips (Figure 6A). The yield of exportable fruits was two and three times higher in plants from heavy slips than in plants from mixed and light slips, respectively (Figure 6C).

In Experiment 2, the type of planting material had no significant effect on the percentage and yield of fruits meeting European export standards (Figures 6B,D).

### Effects of flowering induction at optimum time on average fruit quality attributes

Significant effects of changing from the farmers' flowering induction time to flowering induction at the optimum time were observed in both experiments (Table 5). In cv. Sugarloaf (Experiment 1) infructescence weight and length did not change by inducing flowering at the optimum time, but crown weight and length decreased significantly in all planting material classes and so did fruit length and the crown: infructescence length ratio; fruit weight did not significantly decrease except in plants from light slips (Table 5). Flowering induction at optimum time reduced the proportion of translucent flesh in fruits from light and heavy slips; it also reduced the TSSs in fruits from heavy and mixed slip weights (Table 5).

**Table 5:** **Absolute differences between the average fruit quality, the percentage and yield of fruits meeting European export standards from plants induced at the optimum flowering induction time and those from plants induced at the farmers' flowering induction time and the difference in the number of days between the two flowering induction dates per treatment in Experiments 1 and 2**.

**Experiment and treatment**	**OFI-FFI^a^(days)**	**Quality attributes**									**Percentage exportable fruits ^d^**	**Yield of exportable fruits ^d^**
		**Infructescence weight (kg)**	**Crown weight (kg)**	**Fruit weight (kg)**	**Infructescence length^c^ (cm)**	**Crown length (cm)**	**Fruit length (cm)**	**Ratio^b^ (cm/cm)**	**Translucent flesh (%)**	**TSS^c^ (°Brix)**	**(%)**	**(Mg.ha^−1^)**
**EXPT 1, CV. SUGARLOAF**												
***Slips***												
Light	+57	−0.02ns	−0.12^***^	−0.14^*^	+0.4ns	−8.5^***^	−8.4^**^	−0.71^***^	−13^*^	−0.52 ns	+41.8^***^	+16.6^***^
Mixture of weights	+37	+0.04 ns	−0.11^***^	−0.06 ns	−1.3 ns	−11.6^***^	−12.9^***^	−0.70^**^	+ 12 ns	−1.22^***^	+30.8^*^	+18.4^***^
Heavy	−29	−0.15 ns	−0.03^**^	−0.18 ns	−1.0 ns	−5.5^***^	−6.6^***^	−0.30^*^	−14^**^	−1.19^***^	+13.0 ns	+12.8 ns
**EXPT 2, CV. SMOOTH CAYENNE**												
***Hapas***												
Light	+68	−0.06 ns	+0.00 ns	−0.06 ns	+0.3 ns	+0.5 ns	+0.9 ns	+0.01 ns	+16^*^	−0.17 ns	−1.6 ns	−2.4 ns
Mixture of weights	+55	+0.01 ns	+0.02 ns	+0.04 ns	+0.6 ns	+1.6^*^	+2.2^*^	+0.07 ns	+19^**^	+0.22 ns	+9.5 ns	+8.3 ns
Heavy	+5	−0.28^*^	+0.03^*^	−0.24^*^	−1.8^*^	+0.8 ns	−0.9 ns	+0.10 ns	−14^*^	−1.50^***^	−20.7^***^	−15.5^*^
***Mixture of hapas and ground sucker*s**												
Light	+74	+0.03 ns	−0.01 ns	+0.02 ns	+0.1 ns	−0.1 ns	+0.0 ns	+0.03 ns	+6 ns	+0.43 ns	+0.1 ns	−1.2 ns
Mixture of weights	+50	−0.10 ns	−0.00 ns	−0.11 ns	+1.0 ns	+0.3 ns	+1.3 ns	−0.03 ns	+1 ns	+0.16 ns	−1.5 ns	−2.4 ns
Heavy	+50	−0.06 ns	+0.02 ns	−0.04 ns	−1.3 ns	−0.2 ns	−1.6 ns	+0.03 ns	+10^*^	+0.74^*^	+13.0^*^	+10.6^*^
***Ground suckers***												
Light	+64	−0.12 ns	−0.04^**^	−0.16 ns	−0.8 ns	+1.3 ns	+0.5 ns	+0.15 ns	+26^***^	+0.79 ns	+8.7 ns	+6.0 ns
Mixture of weights	+64	−0.11 ns	−0.02^*^	−0.14 ns	−2.4 ns	−1.4 ns	−3.4^*^	+0.13 ns	+7 ns	+1.04 ns	+6.6 ns	+2.6 ns
Heavy	+15	+0.03 ns	−0.03 ns	+0.00 ns	−0.2 ns	−1.6 ns	−1.9 ns	−0.09 ns	+20^**^	+0.98 ns	+8.7 ns	−2.4 ns

a *OFI, Optimum flowering induction time; FFI, Farmers' flowering induction time*.

b *Ratio, Ratio crown length: infructescence length*.

c *TSS, Total soluble solids*.

d *Fruits meeting European export standards*.

+, An increase in the average quality attributes; −, a decrease in the average quality attributes. Values in bold indicate significant (absolute) differences between the average fruit quality of fruits from plants induced at optimum and fruits from plants induced at farmers' flowering induction time; ns, not statistically significant (P ≥ 0.05);

* statistically significant at 0.05 > P ≥ 0.01;

** statistically significant at 0.01 > P ≥ 0.001;

*** *statistically significant at P < 0.001*.

In Experiment 2, the response depended on the planting material studied and its weight except for the ratio crown: infructescence length that was not affected at all (Table 5). In plants from heavy hapas, induction at optimum time reduced infructescence and fruit weights and slightly increased crown weight (Table 5). In plants from mixed hapas and ground suckers there was no significant change in fruit weight attributes in any of the weight classes (Table 5). In plants from ground suckers induction at the optimum time reduced crown weight in fruits from light and mixed-weight ground suckers and had no effect on fruits from heavy ground suckers (Table 5).

When plants from hapas were induced at optimum time, the infructescence length was reduced in fruits from heavy hapas, but not the crown and fruit lengths. Plants from mixed and light hapas showed an increase in the crown and fruit lengths (Table 5).

In plants from mixed hapas and ground suckers there were no significant changes in infructescence, crown and fruit lengths. When plants from ground suckers were induced at optimum time, only the fruit length was significantly affected: a reduction in fruit length was observed (Table 5).

Flowering induction at optimum time significantly increased the proportion translucent flesh in fruits from light and mixed hapas and reduced it in fruits from heavy hapas. The TSS was only affected in fruits from heavy hapas: a reduction of the TSS was observed (Table 5). In fruits from mixed hapas and ground suckers, only the heavy weight class was significantly affected: an increase of both translucent flesh and TSS was observed. Flowering induction at optimum time significantly increased the translucent flesh in fruits from light and heavy ground suckers, and did not affect significantly the TSS in fruits from any of the ground sucker weight classes (Table 5).

### Effects of flowering induction at optimum time on proportion and yield of fruits meeting European export standards

Flowering induction at optimum time significantly increased the proportion and yield of fruits exportable to Europe in plants from light and mixed-weight slips in Experiment 1, but not significantly in plants from heavy slips (Table 5). Induction at optimum time did not change the proportion or yield of fruits meeting European export standards in plants from light and mixed-weight planting material classes and in heavy ground suckers in Experiment 2 (Table 5), but it reduced the proportion and yield of export-quality fruit in plants from heavy hapas and increased it in plants from the mixture of heavy hapas and heavy ground suckers (Table 5).

## Discussion

### Effects of weight class and type of planting material on average fruit quality attributes

**Figure 7:**
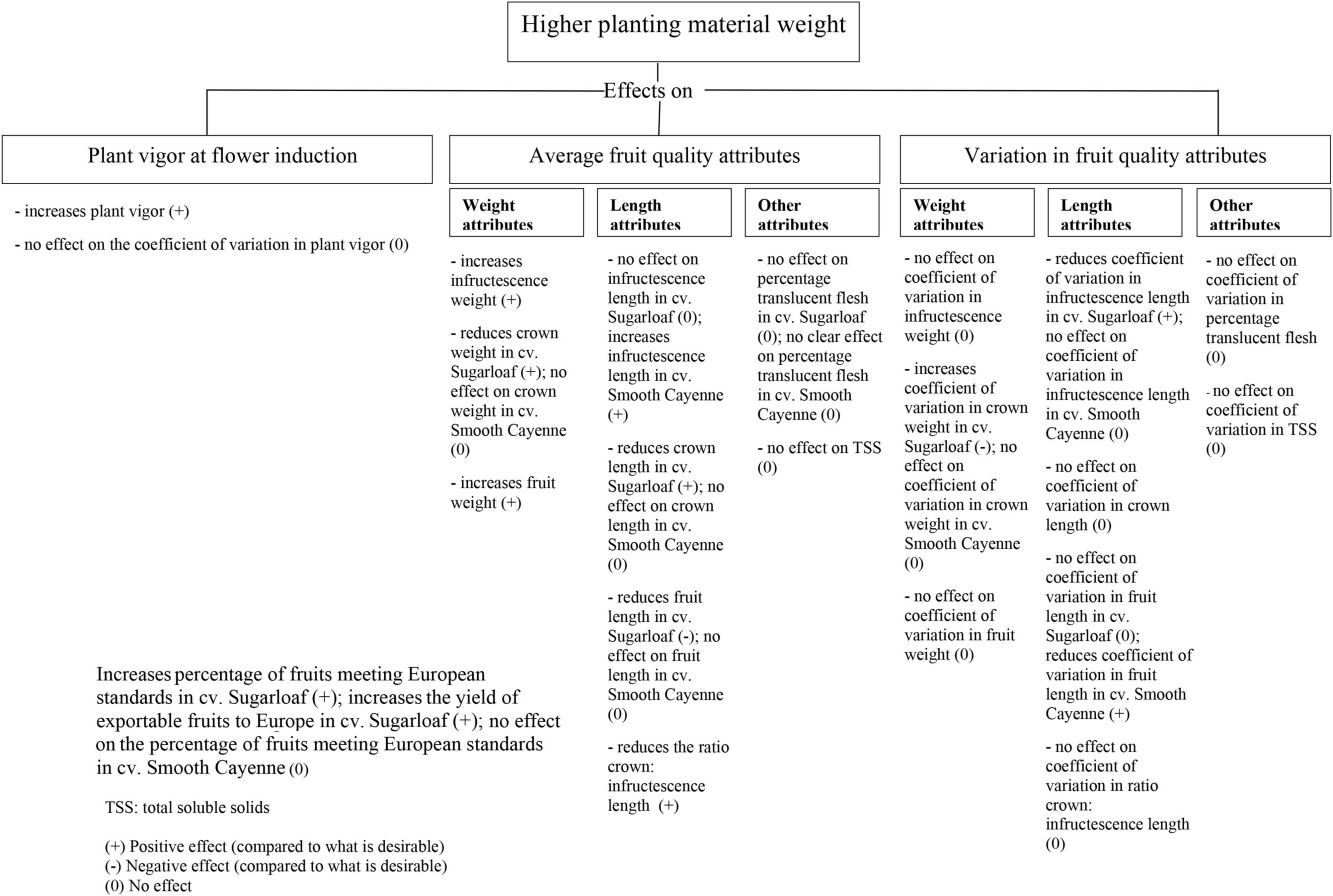
**Effects of higher planting material weight vs. lighter planting material weight in a pineapple crop**.

Results showed that weight class of planting material significantly affected fruit quality (Figure 7). In both experiments, fruits from heavy planting material had heavier infructescence and fruit weights, longer infructescence length, but a shorter crown length and smaller ratio crown: infructescence length than fruits from light planting material (Figure 4). The fact that heavy planting material produced higher fruit weight has been reported by many authors (Mitchell, 1962; Reinhardt et al., 2000; Bhugaloo, 2002) but information on how crown length and the ratio crown: infructescence length are affected have not been reported so far. The findings can be explained by the fact that heavy planting materials will have more reserves at planting; they gave more vigorous plants at flowering induction than light planting materials (Figure 2). The findings are in agreement with those by Fassinou Hotegni et al. (2014b) who found that more vigorous plants (quantified by a higher product of the number of functional leaves × the D-leaf length, as used in the present study) with more assimilates available within a pineapple crop at flowering induction time produced fruits with heavier infructescences and fruits, taller infructescences and a shorter crown and smaller ratio crown: infructescence length.

Fruits from mixed planting material weights showed more or less intermediate average quality between fruits from light and heavy planting material in both experiments (Figure 4), usually being significantly different from either light or heavy planting material, or both. This intermediate quality is in line with their intermediate vigor at the moment of flowering induction (Figure 2). Within a crop, a higher vigor of a pineapple plant at flowering induction time is associated with a higher infructescence and fruit weight, a lower crown weight and crown length and consequently a lower ratio crown: infructescence length (Fassinou Hotegni et al., 2014b). In addition, it is known that most assimilate available at flowering induction is partitioned to the fruit when compared to other parts of the plant like the roots, the stem, and the leaves (Marler, 2011).

Effects of the weight class of planting material on the percentage translucent flesh were not consistent enough to draw appropriate conclusions. The weight class of planting material had no significant effect on TSS. This result is in agreement with that of Bhugaloo (2002) who found that the size of the ground suckers did not affect the TSS.

In Experiment 2, regarding the type of planting material, results show that fruits from hapas had a shorter crown than those from ground suckers (Figure 4J). The presence of roots at planting time might be involved in such differences in crown length. Hapas do not have roots while ground suckers do, because ground suckers are originated below ground on the stem (Hepton, 2003). Such difference in the initial presence of roots between hapas and ground suckers might result in differences in the rate of root production as shown by Ddungu (1971) when using suckers (probably ground suckers), crowns, and slips as planting material. Ddungu (1971) found that the rate of root production in crowns and slips (planting material with no roots at planting time) after planting was higher than that of ground suckers; new root production in the ground suckers occurred after the degenerescence of the old roots reducing the production rate of new roots. In the case of the present study with hapas and ground suckers, and in line with the findings by Ddungu (1971), hapas would have produced more roots than the ground suckers. Also, hapas might produce more leaves at flowering induction time than ground suckers since Norman (1978) showed that planting materials without initial roots at planting (crowns and slips) produced more leaves than suckers. In this study, we did not detect a significant difference between the hapas and ground suckers in vigor of the plants originating from them at flowering induction time (Figure 2), although plants from hapas were slightly more vigorous than those from ground suckers. More vigorous plants at flowering induction leads to fruits with shorter crowns (Fassinou Hotegni et al., 2014b), a possible reason why fruits from hapas showed shorter crowns than those from ground suckers.

The effects of the type of planting material on the fruit weight attributes and other fruit length attributes besides the crown were not significant (Figure 4). The non-significant effects of the type of planting material on the fruit weight and length were in agreement with the findings of Norman (1978) who, in his experiment, used crowns, slips, and ground suckers as planting material.

The type of planting material had no significant effects on the percentage of translucent flesh and TSS in Experiment 2 (Figure 5). This suggests that the sugar concentration in the fruit is independent of the type of planting material when hapas and ground suckers are used.

### Effects of weight and type of planting material on variation in fruit quality attributes

In this study, we primarily aimed at evaluating the effects of the weight class and type of planting material on the variation in fruit quality, expecting a larger fruit quality variation in mixed-weight classes (Experiment 1 and 2) and mixed planting material types (Experiment 2). Surprisingly, the results indicated that effects of weight class and the type of planting material on the variation (expressed by the CV) in fruit quality attributes were not significant except some inconsistent significant effects of the weight class on the CV in crown weight and infructescence length in Experiment 1 (Table 3) and in fruit length in Experiment 2 (Table 3). The initial variation in weight of planting material might have been partly compensated during crop development as shown by the *P*-values for CV for vigor of the plants at the moment of flowering induction already being not significant, although low (Table 2). Uncontrolled factors such as differences in soil conditions within a field might have played a role. Especially in long duration crops like pineapple these may have a large effect on variation. Incidental effects of the weight of planting material on the variation in crown weight and infructescence length were reflected by fruits from plants from heavy slips showing higher CV in crown weight and lower CV in infructescence length than fruits from mixed and light slips (Tables 3, 4).

In Experiment 2, the type of planting material had no effect on the CV in the different quality attributes except on the CV in TSS (Tables 3, 4). It was expected that using a mixture of hapas and ground suckers would lead to a higher CV in most quality attributes compared with when a single type of planting material was used. This again suggests that hapas and ground suckers in cv. Smooth Cayenne hardly differed in performance.

### Effects of weight and type of planting material on percentage and yield of fruits meeting European export standards

Plants from heavy slips gave more fruits meeting European export standards than plants from other weights classes in Experiment 1 (Figure 6). This was mainly due to the fact that fruits from heavy planting material had smaller crowns (Figure 4I), taller infructescences (Figure 4G) and consequently a shorter ratio crown: infructescence length (Figure 4M) than fruits from other weights classes. Also the total exportable fruit yield in plants from heavy slips was 26 and 19 Mg.ha^−1^ higher than that of fruits from light and mixed slips, respectively. This could be explained by the improvement in the number of fruits meeting the exporting criteria. The weight of planting material had no effect on the percentage of fruits exportable to Europe in Experiment 2. This implies that the improvement in fruit weight and mainly in ratio crown length: infructescence length in fruits from heavy planting material (Figures 4F,N) was not enough to affect significantly the proportion of fruits exportable to Europe.

The type of planting material (hapas or ground suckers) used to grow cv. Smooth Cayenne in Experiment 2 had no significant effect on the proportion of fruits meeting European export standards (Figure 6B) because the average quality attribute was not affected in most quality attributes.

### Effects of induction at optimum time on average fruit quality attributes and proportion and yield of fruits meeting European export standards

In Experiment 1, flowering induction at optimum time reduced crown weight and length, fruit length and the ratio crown: infructescence length in cv. Sugarloaf (Table 5). These effects might be due to the time elapsing between the optimum induction time and the farmers' flowering induction time (Table 5), i.e., +57 days for plants from light slips; +37 days for plants from the mixture of slips; and −29 days for plants from heavy slips. During that period of time (when positive) the plant will continue its growth producing new leaves and consequently increasing its vigor before the flowering induction time. The negative value obtained in plants from heavy planting material suggests the farmers' flowering induction time, i.e., 12 months after planting (Table S1) was too late for cv. Sugarloaf grown from heavy slips. The reduction in fruit length was the consequence of the reduction in the crown length since the infructescence length was not affected by flowering induction time (Table 5). Flowering induction at optimum time did not affect the infructescence weight. Reduction in fruit weight was found in fruits from plants from light slips (Table 5); this reduction was due especially to the reduction in the crown weight. Flowering induction at optimum time increased the proportion and yield of fruits meeting European export standards in plants from light and mixed slip weight intervals in cv. Sugarloaf (Table 5). This might be due to the significant reduction in the crown: infructescence length ratio (Table 5). The fruit weight was hardly affected (Table 5).

In cv. Smooth Cayenne in Experiment 2, very limited effects of the change from the flowering induction at the farmers' flowering induction time to the induction at the optimum time on the average fruit weight and length attributes quality were observed (Table 5); in addition it was found that flowering induction of cv. Smooth Cayenne at optimum time only increased the proportion and yield of fruits exportable to Europe in fruits from a mixture of heavy hapas plus ground suckers (Table 5). This implies that in the other weights classes, other quality attributes were limiting the proportion of fruits meeting European export standards. The inconsistent trend in the reduction or increase in flesh translucency and the TSS caused by the induction at optimum induction time might be due to different temperature conditions, shown by Paull and Reyes (1996) to affect the proportion translucent flesh in pineapple and by Pessarakli (2001) to affect the TSS in grape fruits.

## Conclusions

The study revealed that weight of planting material affected the fruit quality attributes. In both experiments, fruits from plants from heavy planting material had heavier infructescence and fruit weights, longer infructescence length, shorter crown length and smaller ratio crown: infructescence length than fruits from light planting material. So far no literature has reported such differences in the individual infructescence and crown attributes caused by the weight of planting material used. When hapas or ground suckers were used as planting material, the type of planting material did not affect the average fruit quality attributes except the crown length which was shorter in fruits from hapas than in those from ground suckers. The weight and type (hapas or ground suckers) of planting material had in general limited or no effects on the variation in fruit quality attributes except for some incidental effects found in few quality attributes.

Plants from heavy slips gave more fruits and a higher yield of fruits that were exportable to Europe than plants from other slip weight classes in cv. Sugarloaf. When considering the hapas, ground suckers, and the mixture of hapas and ground suckers in cv. Smooth Cayenne, the weight and type of planting material had no effect on the proportion and yield of fruits exportable to Europe. Flowering induction at optimum time increased the proportion and the yield of fruits meeting European export standards in light and mixed slip weight classes in cv. Sugarloaf due to a strong decrease in the ratio crown: infructescence length. In cv. Smooth Cayenne, flowering induction of the plants from the mixture of heavy hapas and heavy ground suckers at optimum time increased the proportion of fruits exportable to Europe due to the increase in the TSSs. The knowledge brought by this study is important to design appropriate cultural practices to produce higher quality pineapple fruits.

### Conflict of interest statement

The authors declare that the research was conducted in the absence of any commercial or financial relationships that could be construed as a potential conflict of interest.

## Abbreviations

CV: Coefficient of variation
TSS: Total soluble solids.
