# Supplementary material for: Influence of weight and type of planting material on fruit quality and its heterogeneity in pineapple [Ananas comosus (L.) Merrill]
Source: Front Plant Sci. 2015 Jan 21;5:798. doi: 10.3389/fpls.2014.00798 (PMC4300867; doi:10.3389/fpls.2014.00798)
Supplement: Supplementary file 1 [file DataSheet1.PDF]

# *Supplementary Material*

# Influence of weight and type of planting material on fruit quality and its heterogeneity in pineapple [*Ananas comosus* (L.) Merrill]

V.N. Fassinou Hotegni[1,2], W.J.M. Lommen[1*], E.K. Agbossou[2] and P.C. Struik[1]

[1] Centre for Crop Systems Analysis, Wageningen University, Wageningen, the Netherlands
[2] Faculté des Sciences Agronomiques, Université d'Abomey Calavi, Cotonou, Benin,

***Correspondence:** Dr. Willemien J.M. Lommen, Centre for Crop Systems Analysis, Wageningen University, 6708 PB Wageningen, the Netherlands
willemien.lommen@wur.nl

## 1. Supplementary Table

Table S1.

**Table S1** Information on flowering induction and harvesting times in the two experiments with cvs Sugarloaf or Smooth Cayenne

| | Experiment 1, cv. Sugarloaf | Experiment 2, cv. Smooth Cayenne |
|---|---|---|
| Planting time | 24 February 2012 | 9 November 2011 |
| **Farmers' practice** | | |
| Farmers' flowering induction time[a] | 18 February 2013 (360 DAP[b]) | 5 November 2012 (362 DAP) |
| Harvesting time | | |
| *Slips* | | |
| Light | 19, 20 July 2013 (511, 512 DAP) | -[c] |
| Mixture of weights | 19, 20 July 2013 (511, 512 DAP) | - |
| Heavy | 18, 19, 21 July 2013 (510, 511, 513 DAP) | - |
| *Hapas* | | |
| Light | - | 8 April 2013 (516 DAP) |
| Mixture of weights | - | 8 April 2013 (516 DAP) |
| Heavy | - | 8 April 2013 (516 DAP) |
| *Hapas + ground suckers* | | |
| Light | - | 8 April 2013 (516 DAP) |
| Mixture of weights | - | 8 April 2013 (516 DAP) |
| Heavy | - | 8 April 2013 (516 DAP) |
| *Ground suckers* | | |
| Light | - | 8 April 2013 (516 DAP) |
| Mixture of weights | - | 8 April 2013 (516 DAP) |
| Heavy | - | 8 April 2013 (516 DAP) |
| **Optimum practice** | | |
| Optimum flowering induction time | | |
| *Slips* | | |
| Light | 16 April 2013 (417 DAP) | - |
| Mixture of weights | 27 March 2013 (397 DAP) | - |
| Heavy | 20 January 2013 (331 DAP) | - |
| *Hapas* | | |
| Light | - | 12 January 2013 (430 DAP) |
| Mixture of weights | - | 30 December 2012 (417 DAP) |
| Heavy | - | 10 November 2012 (367 DAP) |

**Table S1** Continued

| | Experiment 1, cv. Sugarloaf | Experiment 2, cv. Smooth Cayenne |
|---|---|---|
| Optimum flowering induction time | | |
| *Hapas + ground suckers* | | |
| Light | - | 8 January 2013 (426 DAP) |
| Mixture of weights | - | 8 January 2013 (426 DAP) |
| Heavy | - | 20 November 2012 (377 DAP) |
| *Ground suckers* | | |
| Light | - | 18 January 2013 (436 DAP) |
| Mixture of weights | - | 25 December 2012 (412 DAP) |
| Heavy | - | 25 December 2012 (412 DAP) |
| Harvesting time | | |
| *Slips* | | |
| Light | 17, 19 and 20 Sept. 2013 (571, 573, 574 DAP) | - |
| Mixture of weights | 26, 27 and 28 Aug. 2013 (549, 550 , 551 DAP) | - |
| Heavy | 21, 22 and 24 June 2013 (483, 484, 486 DAP) | - |
| *Hapas* | | |
| Light | - | 15 June 2013 (584 DAP) |
| Mixture of weights | - | 2 June 2013 (571 DAP) |
| Heavy | - | 13 April 2013 (521 DAP) |
| *Hapas + ground suckers* | | |
| Light | - | 11 June 2013 (580 DAP) |
| Mixture of weights | - | 11 June 2013 (580 DAP) |
| Heavy | - | 23 April 2013 (531 DAP) |
| *Ground suckers* | | |
| Light | - | 21 June 2013 (590 DAP) |
| Mixture of weights | - | 28 May 2013 (566 DAP) |
| Heavy | - | 28 May 2013 (566 DAP) |

[a]: All treatments have been induced; [b] DAP: days after planting; [c]: not applicable because of the type of planting material
