# Supplementary material for: Influence of weight and type of planting material on fruit quality and its heterogeneity in pineapple [Ananas comosus (L.) Merrill]
Source: Front Plant Sci. 2015 Jan 21;5:798. doi: 10.3389/fpls.2014.00798 (PMC4300867; doi:10.3389/fpls.2014.00798)
Supplement: Supplementary file 2 [file DataSheet2.PDF]

Supplementary Material

Influence of weight and type of planting material on fruit quality and its heterogeneity in pineapple [*Ananas comosus* (L.) Merrill]

V.N. Fassinou Hotegni[1,2], W.J.M. Lommen[1*], E.K. Agbossou[2] and P.C. Struik[1]

[1] Centre for Crop Systems Analysis, Wageningen University, Wageningen, the Netherlands
[2] Faculté des Sciences Agronomiques, Université d'Abomey Calavi, Cotonou, Benin,

***Correspondence:** Dr. Willemien J.M. Lommen, Centre for Crop Systems Analysis, Wageningen University, 6708 PB Wageningen, the Netherlands
willemien.lommen@wur.nl

1. Supplementary Figures

Figures S1, S2, S3, S4, S5, S6, S7, S8 and S9.

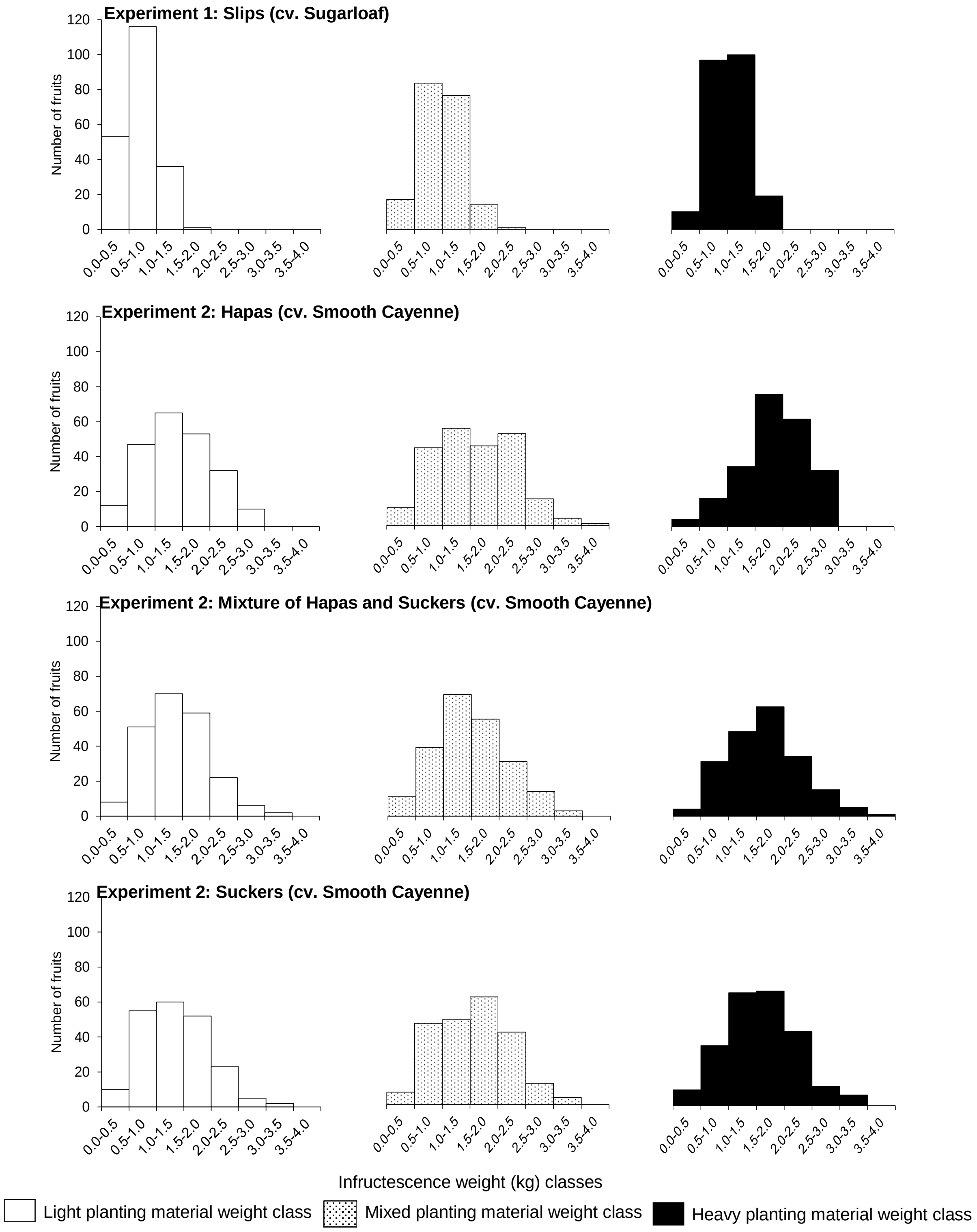

Figure S1 Frequency distribution of the infructescence weight (kg) in plants induced at farmer's flowering induction time, as affected by weight class (Experiments 1 and 2) and type of planting material (Experiment 2)

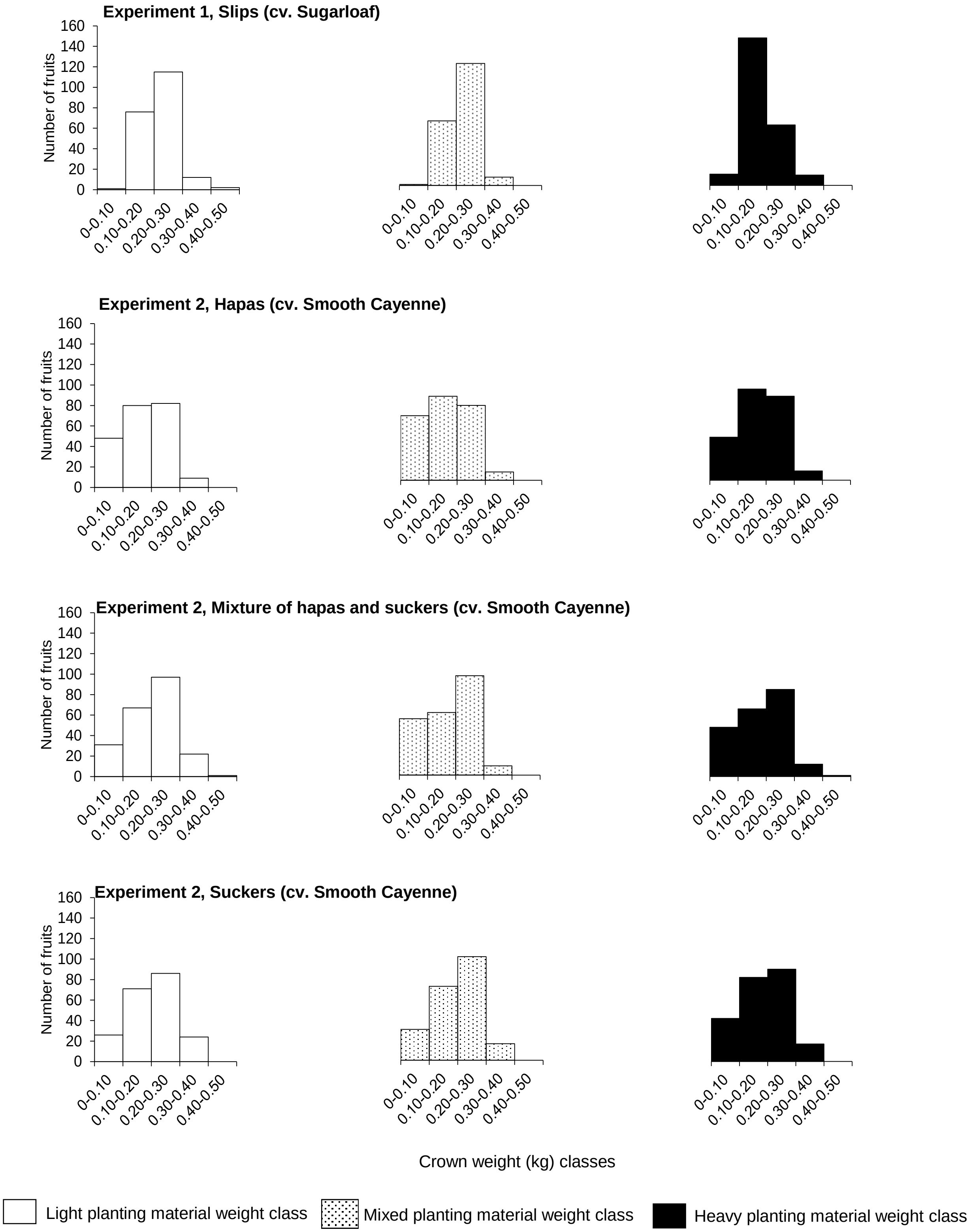

Figure S2 Frequency distribution of the crown weight (kg) in plants induced at farmer's flowering induction time, as affected by weight class (Experiments 1 and 2) and type of planting material (Experiment 2)

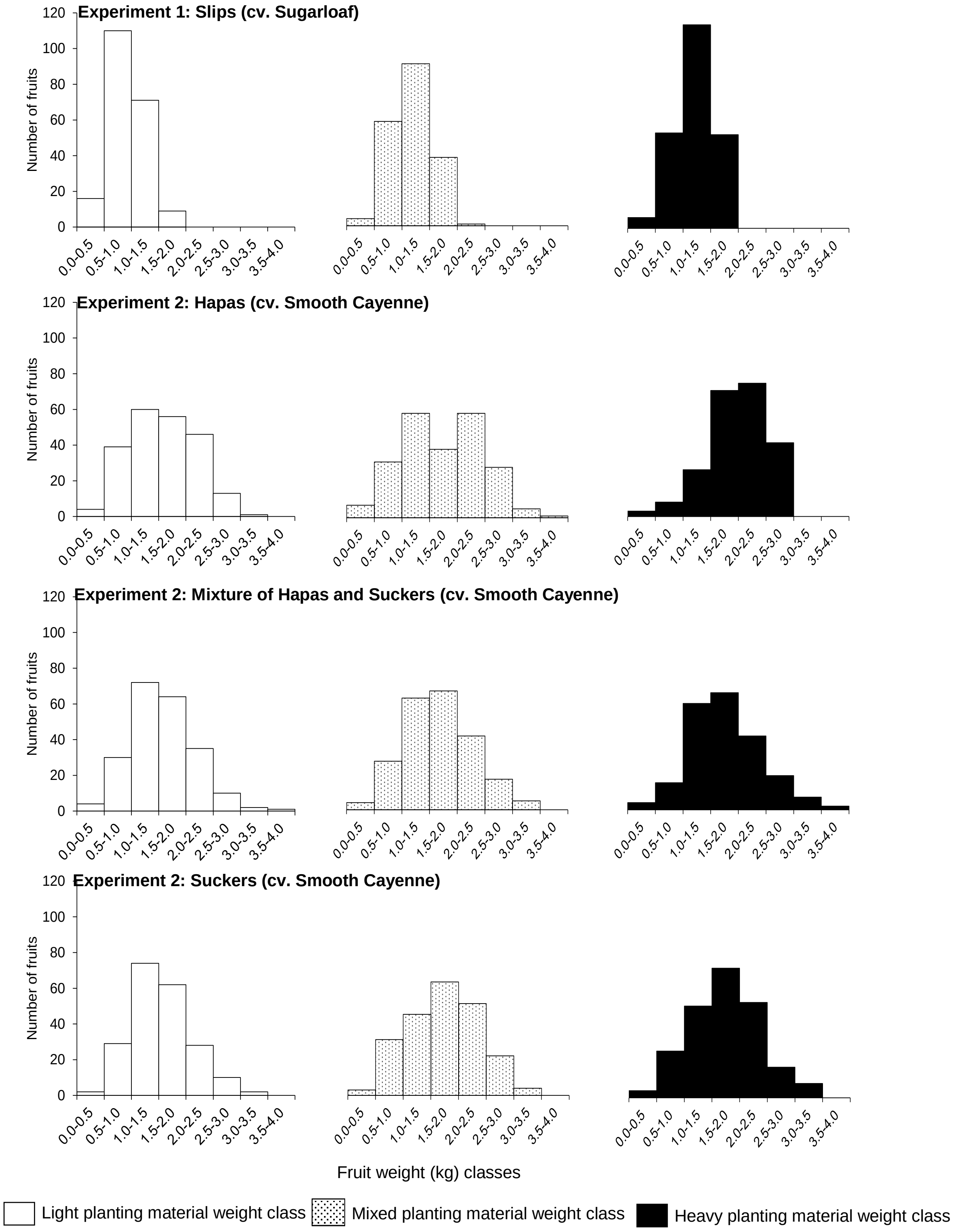

Figure S3 Frequency distribution of the fruit weight (kg) in plants induced at farmer's flowering induction time, as affected by weight class (Experiments 1 and 2) and type of planting material (Experiment 2)

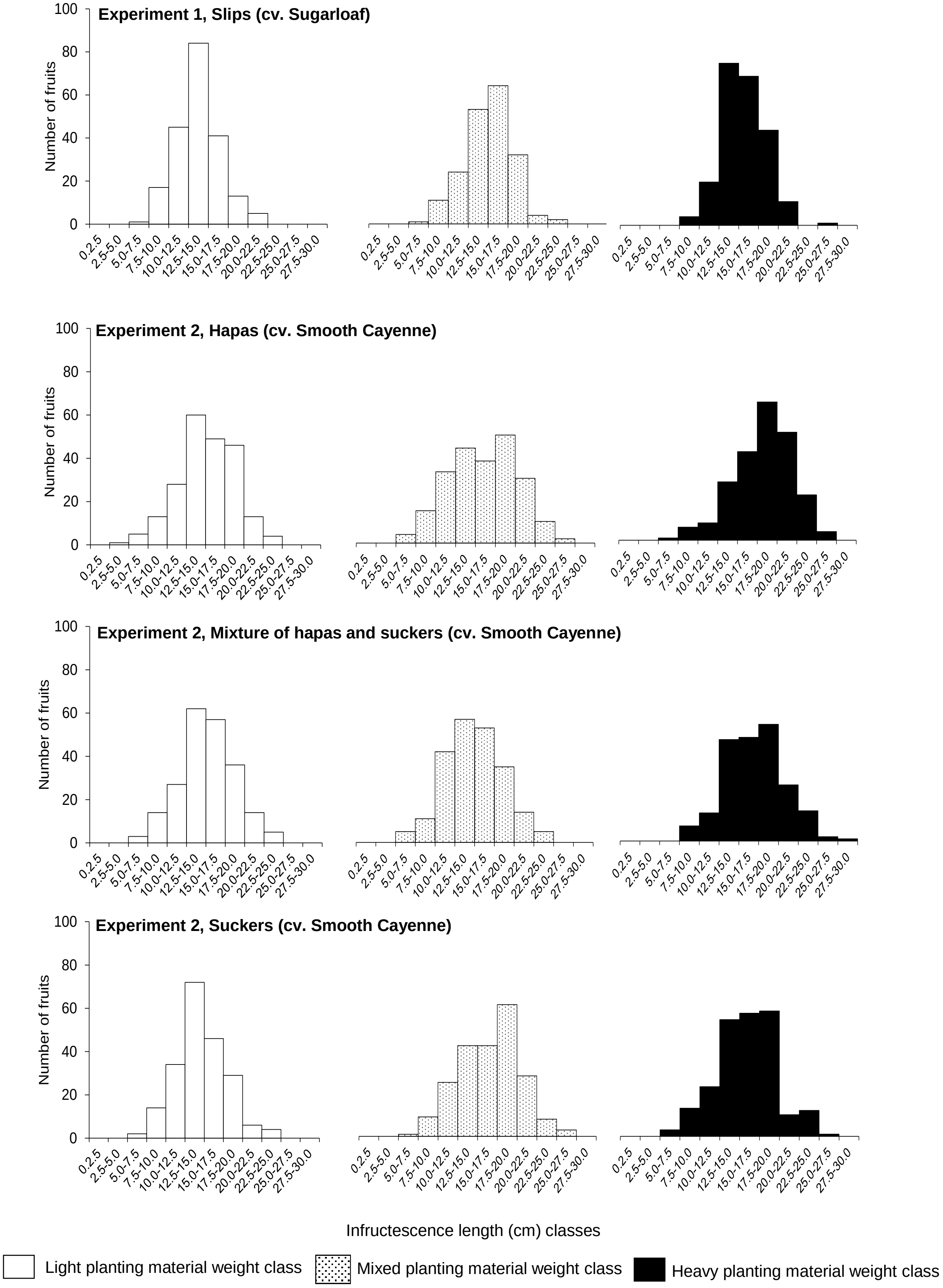

Figure S4 Frequency distribution of the infructescence length in plants induced at farmer's flowering induction time, as affected by weight class (Experiments 1 and 2) and type of planting material (Experiment 2)

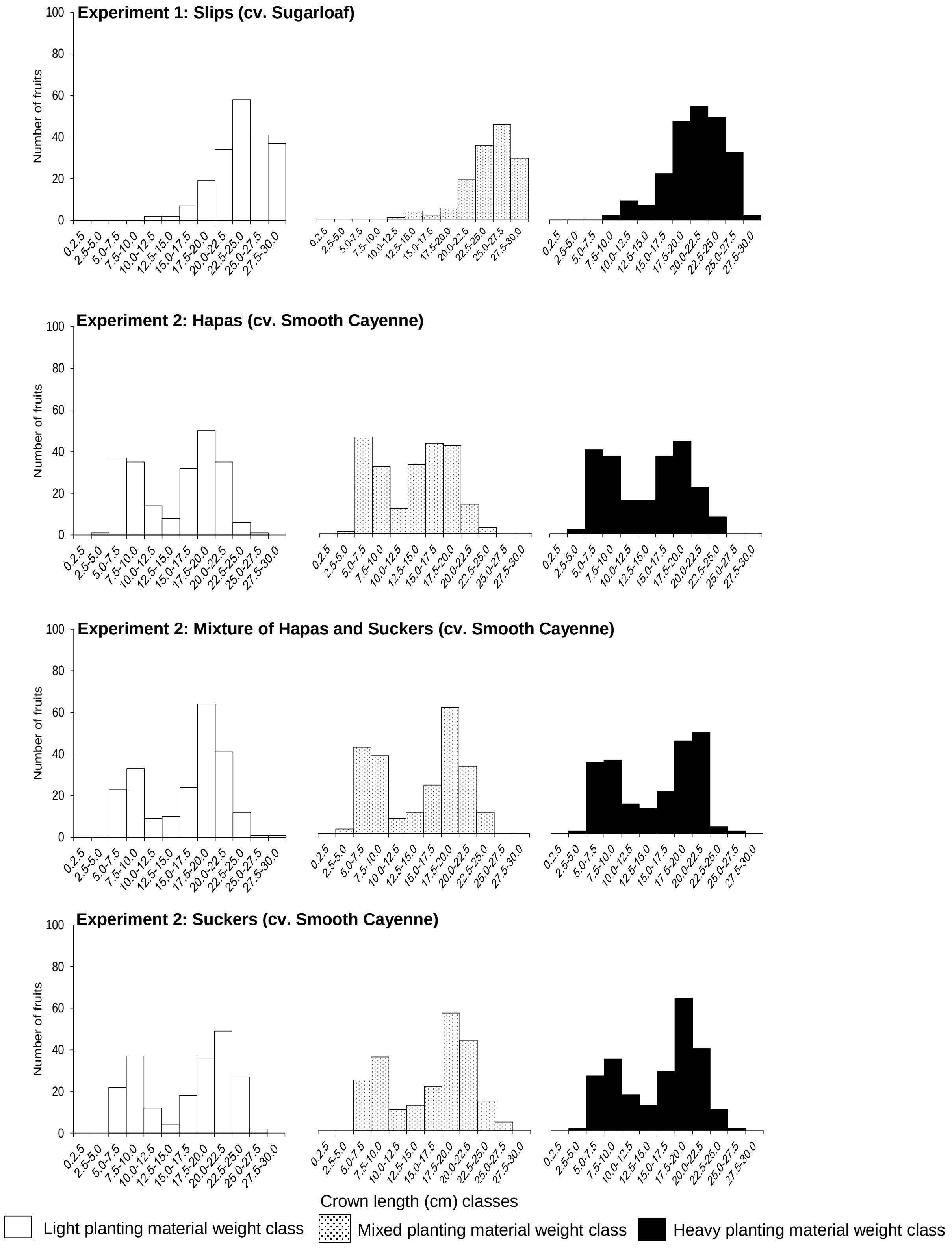

Figure S5 Frequency distribution of the crown length in plants induced at farmer's flowering induction time, as affected by weight class (Experiments 1 and 2) and type of planting material (Experiment 2)

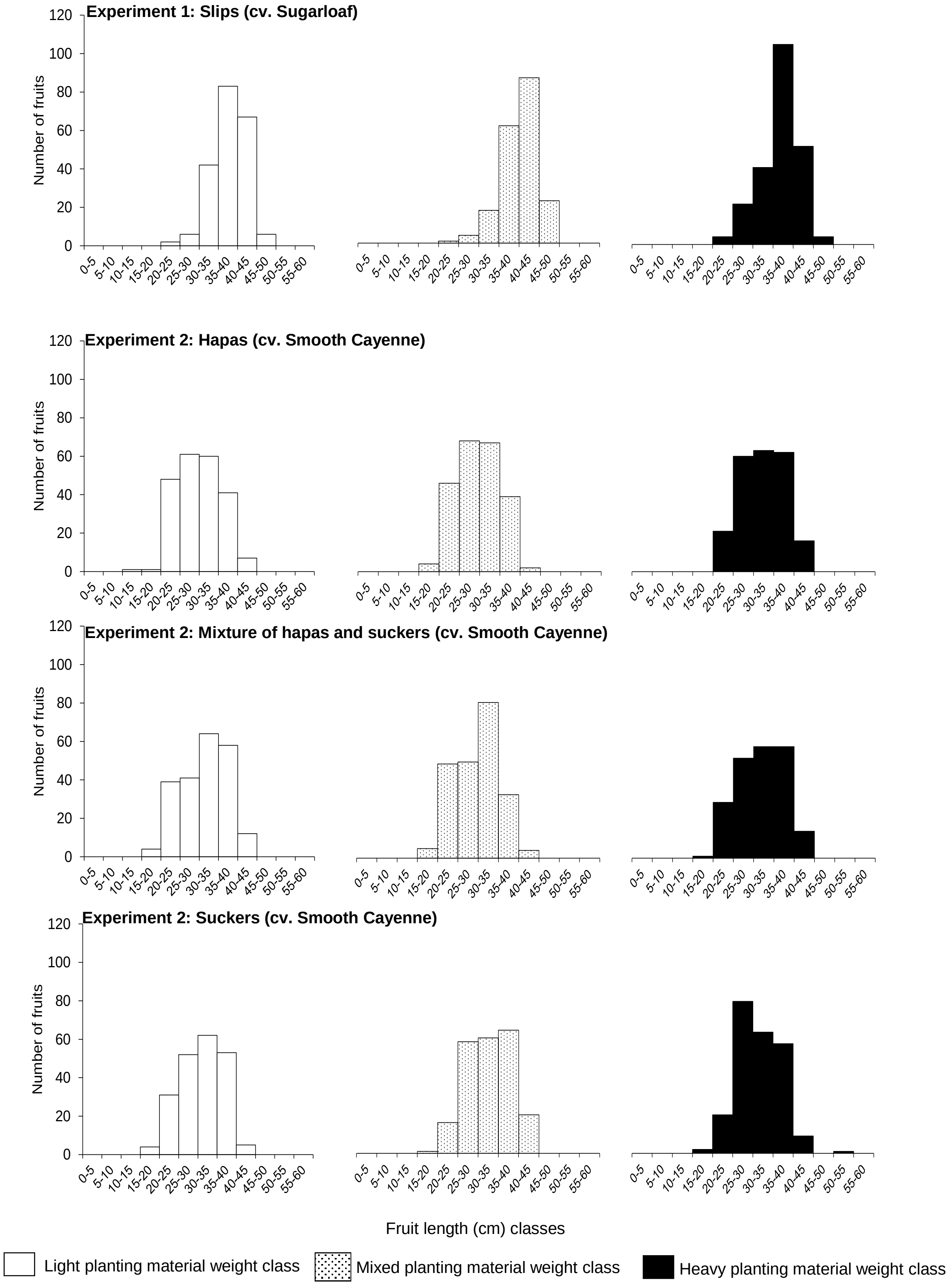

Figure S6 Frequency distribution of the fruit length in plants induced at farmer's flowering induction time, as affected by weight class (Experiments 1 and 2) and type of planting material (Experiment 2)

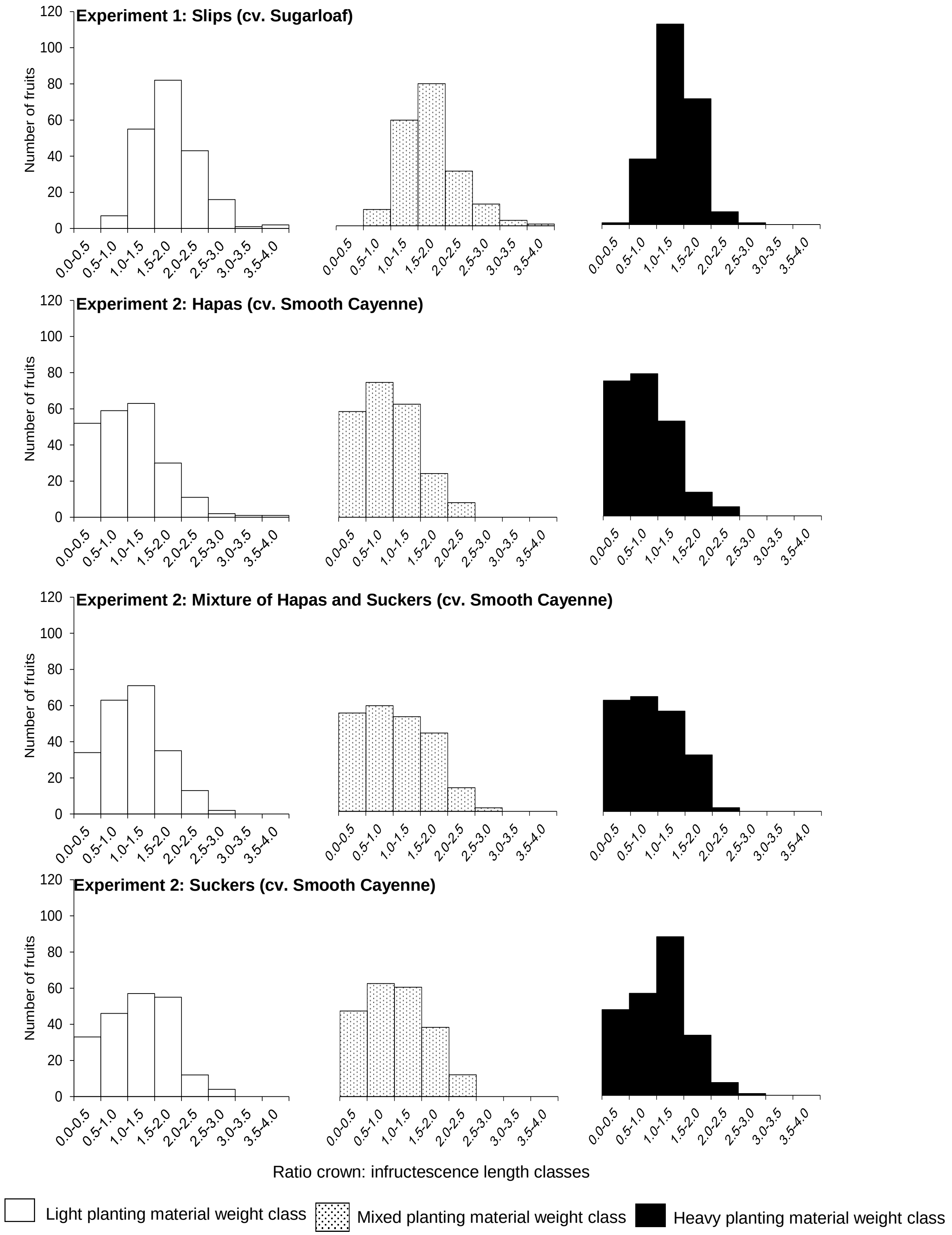

Figure S7 Frequency distribution of the ratio crown: infructescence length in plants induced at farmer's flowering induction time, as affected by weight class (Experiments 1 and 2) and type of planting material (Experiment 2)

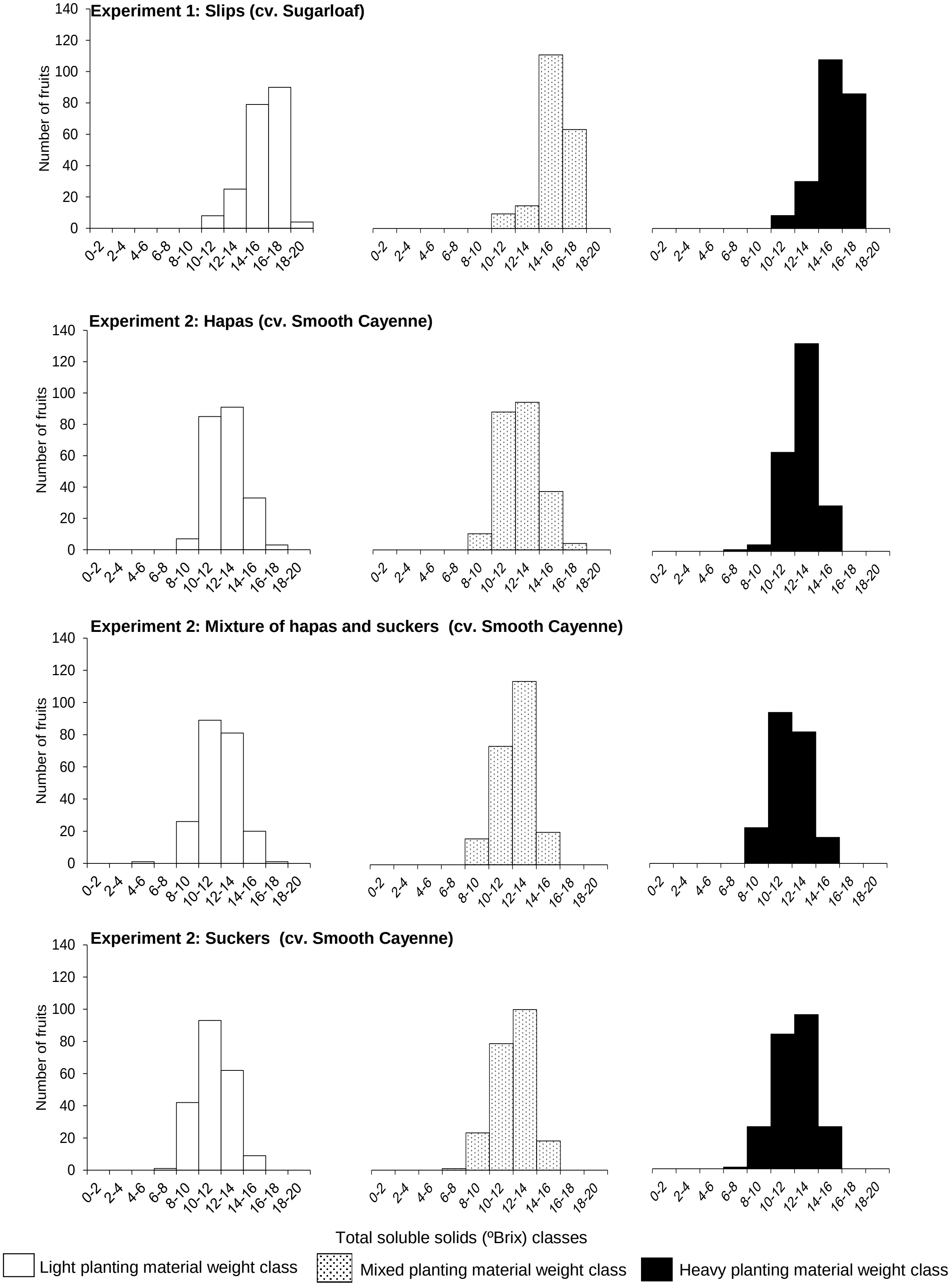

Figure S8 Frequency distribution of the total soluble solids in plants induced at farmer's flowering induction time, as affected by weight class (Experiments 1 and 2) and type of planting material (Experiment 2)

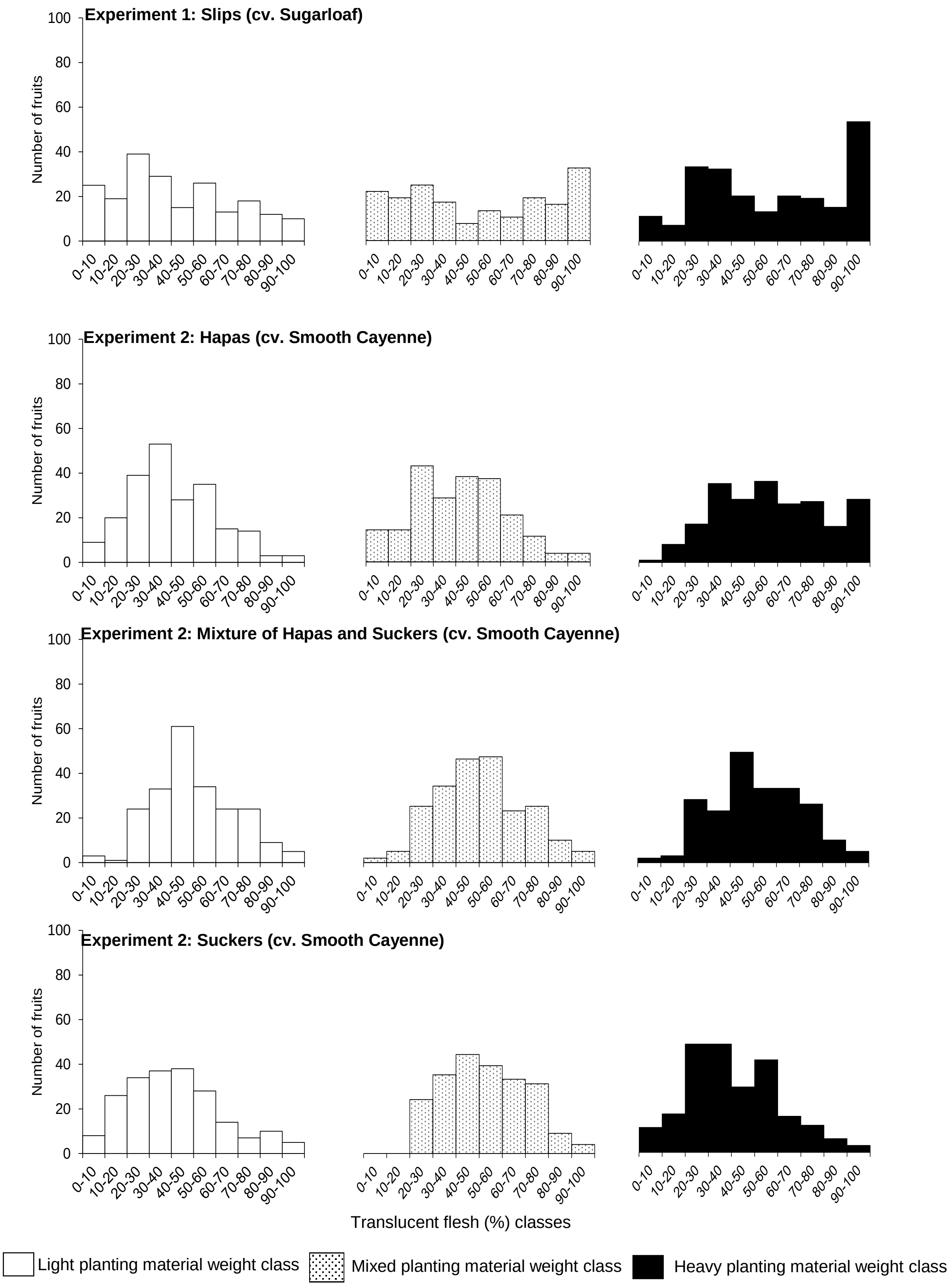

Figure S9 Frequency distribution of the translucent flesh in plants induced at farmer's flowering induction time, as affected by weight class (Experiments 1 and 2) and type of planting material (Experiment 2)
